# Small, synthetic, GC-rich mRNA stem-loop modules 5′ proximal to the AUG start-codon predictably tune gene expression in yeast

**DOI:** 10.1186/1475-2859-12-74

**Published:** 2013-07-29

**Authors:** Erwin Lamping, Masakazu Niimi, Richard D Cannon

**Affiliations:** 1 Sir John Walsh Research Institute, University of Otago, Dunedin 9054, New Zealand

**Keywords:** mRNA stem-loops, Post-transcriptional regulation of gene expression, Inhibition of translation initiation, Yeast 43S preinitiation complex, Regulation of translation, Negative interactions of multiple cloning-sites

## Abstract

**Background:**

A large range of genetic tools has been developed for the optimal design and regulation of complex metabolic pathways in bacteria. However, fewer tools exist in yeast that can precisely tune the expression of individual enzymes in novel metabolic pathways suitable for industrial-scale production of non-natural compounds. Tuning expression levels is critical for reducing the metabolic burden of over-expressed proteins, the accumulation of toxic intermediates, and for redirecting metabolic flux from native pathways involving essential enzymes without negatively affecting the viability of the host. We have developed a yeast membrane protein hyper-expression system with critical advantages over conventional, plasmid-based, expression systems. However, expression levels are sometimes so high that they adversely affect protein targeting/folding or the growth and/or phenotype of the host. Here we describe the use of small synthetic mRNA control modules that allowed us to predictably tune protein expression levels to any desired level. Down-regulation of expression was achieved by engineering small GC-rich mRNA stem-loops into the 5′ UTR that inhibited translation initiation of the yeast ribosomal 43S preinitiation complex (PIC).

**Results:**

Exploiting the fact that the yeast 43S PIC has great difficulty scanning through GC-rich mRNA stem-loops, we created yeast strains containing 17 different RNA stem-loop modules in the 5′ UTR that expressed varying amounts of the fungal multidrug efflux pump reporter Cdr1p from *Candida albicans*. Increasing the length of mRNA stem-loops (that contained only GC-pairs) near the AUG start-codon led to a surprisingly large decrease in Cdr1p expression; ~2.7-fold for every additional GC-pair added to the stem, while the mRNA levels remained largely unaffected. An mRNA stem-loop of seven GC-pairs (∆G = −15.8 kcal/mol) reduced Cdr1p expression levels by >99%, and even the smallest possible stem-loop of only three GC-pairs (∆G = −4.4 kcal/mol) inhibited Cdr1p expression by ~50%.

**Conclusion:**

We have developed a simple cloning strategy to fine-tune protein expression levels in yeast that has many potential applications in metabolic engineering and the optimization of protein expression in yeast. This study also highlights the importance of considering the use of multiple cloning-sites carefully to preclude unwanted effects on gene expression.

## Background

Recent advances in synthetic biology and bioinformatics together with exponentially growing biological databases and the -omics revolution, especially transcriptomics and metabolomics, have increased the importance of yeast to industrial biotechnology [1,2]. *Saccharomyces cerevisiae* is a key eukaryotic model organism for fundamental molecular biology research, it was the first eukaryotic organism to have its entire genome sequenced [3], and it is also a common industrial microorganism used extensively in food and beverage production. These factors, together with its genetic tractability and its ability to grow at low pH, have made *S. cerevisiae* an attractive microorganism to be used as a chemical factory. Gibson *et al.*, 2008, have assembled the entire *Mycoplasma genitalium* genome in yeast [4], and Shao *et al.*, 2009, used transformation-associated recombination to assemble entire metabolic pathways in one single step in yeast [5]. The list of non-natural biological compounds successfully produced by *S. cerevisiae* is diverse and ranges from protein drugs to fine and commodity chemicals [1], advanced biofuels [6], the large family of benzylisoquinoline alkaloids [7] and many other secondary metabolites with a wide range of pharmacological activities [2] including the successful production of high levels of artemisinin [8], a highly effective antimalarial.

Despite these significant advances in synthetic biology major challenges in the design of optimal metabolic pathways remain. To obtain maximal yield, pathway flux needs to be optimized, and the accumulation of toxic intermediates and the metabolic burden on the host minimized. Therefore, one of the key challenges of pathway engineering is the regulation of individual pathway enzymes for optimal activity [2]. Control of expression still relies heavily on regulatable, often plasmid-based, expression systems, but their use is largely limited to research and development only. Both regulatable promoters and plasmids require expensive synthetic media for their stable maintenance and controlled function (i.e. addition of inducers or repressors). In addition, many inducers exhibit disadvantageous pleiotropic effects [9,10] that affect other aspects of the cell’s biology and/or physiology that are often not well characterized and may lead to misinterpretations of the induced effects or negatively affect expression of foreign genes. Thus, an ideal production host requires expression modules stably integrated into the genome with each enzyme expression level individually optimized in a way that does not depend on regulatable promoters and the use of complex synthetic media. Alper *et al.*, 2005, provided an elegant solution by creating constitutive promoter libraries in *Escherichia coli* and *S. cerevisiae* that drove a wide (~1000-fold) dynamic range of protein production [11,12]. However, the lack of well-characterized promoters still provides a significant hurdle for pathway engineering in yeast.

Here we describe how we discovered a way to tune protein production predictably in yeast. This was revealed during the development of a novel system for the constitutive expression of exceptionally high levels of functional heterologous membrane proteins in *S. cerevisiae*. The expression system consists of plasmid pABC3 and derivative plasmids and the *S. cerevisiae* hosts AD1-8u^-^ and its close relative AD∆ [13,14]. Both strains are deleted in seven ABC transporters, which makes them exquisitely sensitive to a wide range of xenobiotics [15,16], and the transcription factor *PDR3*. They also contain the gain-of-function mutant transcription factor Pdr1-3p, that drives the hyper-expression of heterologous ORFs from single-copy genes stably integrated at the genomic *PDR5* locus [13-15,17]. This system has several advantages over other, plasmid-based, expression systems: i) significant cost savings for large-scale protein production, as there is no need for expensive synthetic media for the maintenance of plasmids; ii) robust, highly reproducible, phenotypes and homogenous cell populations; and iii) improved homogeneity of the expressed protein. The objective of this study was to develop a strategy to down-regulate these constitutively over-expressed proteins in a way that avoids the potential disadvantages of existing regulatory systems. When we tried to improve the cloning efficiency of large (7~kb) expression modules by replacing all hexamer cutting sites of the multiple cloning-site (MCS) of pSK-PDR5-PPUS [15] with rare 8 bp cutting sites we noticed that inclusion of the GC-rich *Sfi*I site reduced protein expression levels ~8-fold. This forced us to use a single AT-rich 8 bp cutting site (*Pac*I) for efficient cloning of the 5′ end of heterologous ORFs in plasmid pABC3 [14] - thus indicating, as shown by Crook *et al.*, 2011 [18], that MCSs can be far from benign cloning tools.

Investigation of AUG start-codon scanning has shown that the yeast ribosomal 43S preinitiation complex (PIC) is very sensitive to interfering hairpins in their 5′ UTRs [19-24], which, as will be demonstrated in this article, explains why the GC-rich *Sfi*I site was so detrimental for protein production. Although it has long been established that mRNA stem-loops in the 5′ UTR near the AUG start codon inhibit protein expression in eukaryotes, and their inhibitory activities appeared largely independent of gene context [19,21,22,24], no attempts have been made to exploit this intrinsic feature of the eukaryotic translation machinery to regulate protein production in yeast. The only systematic study of the effects of the stability, size, sequence and position of a set of different mRNA stem-loop constructs, expressed in COS-7 cells, found that stems of identical stability but with increasing GC content from (52% to 92%) diminished the expression of a GFP reporter by over 18-fold [25] indicating that it is not only the thermodynamic stability of the stem-loop *per se* but also, and perhaps more importantly, its GC-content that determines its degree of inhibition of protein expression/translation in mammalian cells.

In this study we created 17 systematically modified mRNA stem-loop constructs in front of the *C. albicans* multidrug efflux pump reporter Cdr1p ORF which revealed minimal features necessary for effective repression of protein expression in yeast. Stem-loops of mixed A/U- and G/C-pair containing stems inhibited Cdr1p expression less predictably. However, Cdr1p expression controlled by mRNA stem-loops comprising stems containing only GC-pairs was highly predictable and decreased exponentially with the number of GC-pairs in the stem. Even the smallest stem-loop stem of 3 GC-pairs inhibited Cdr1p expression by ~50%. Additional fine-tuning of expression could be achieved by varying the size of the loop. The degree of translation inhibition by individual mRNA stem-loop modules appeared an intrinsic feature that was independent of: i) sequence context; ii) the host yeast strain; iii) the growth medium; iv) the pH and carbon source of the growth medium; v) steady-state mRNA levels; and vi) they also appeared independent of the growth stage of cells. The stable and predictable tuning of protein expression, with a large dynamic range, from a single promoter by well-defined, small, GC-rich mRNA stem-loops near the AUG start codon provides a simple and very powerful tool for optimal pathway engineering and synthetic biology in yeast. It also provides important clues for an improved understanding of the molecular mechanism of AUG start codon scanning of the yeast 43S PIC.

## Results

### An *Sfi*I cloning site 5′ proximal to the ATG start-codon severely affects gene expression levels in yeast

**Figure 1:**
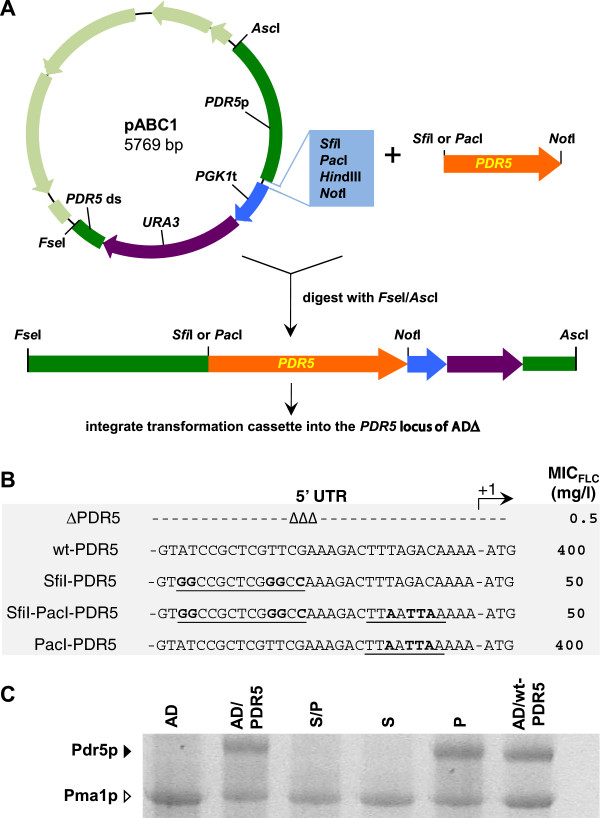
**The *Sfi*I restriction enzyme cloning site severely inhibits gene expression in yeast. A.** A schematic diagram of the cloning strategy used to over-express heterologous ORFs in *S. cerevisiae*. *PDR5* (orange) was cloned as *Sfi*I/*Not*I or *Pac*I/*Not*I fragments into pABC1 (the pBluescriptIISK(+) vector backbone is light green and the multiple cloning site of the transformation cassette light blue). pABC1-PDR5 was digested with *Fse*I and *Asc*I to release the 7.4 kb transformation cassette [*PDR5* promoter (green)-ORF (orange)-*PGK1* terminator (blue)-*URA3* marker (purple)-*PDR5* downstream region (green)]. The transformation cassettes were gel purified and used to transform *S. cerevisiae* AD to Ura^+^. **B.** Effect of 5′ UTR on Pdr5p activity. Strains expressing Pdr5p were created either using pABC1 (SfiI-PDR5, SfiI-PacI-PDR5) or pABC3 (PacI-PDR5), as previously described [14], and ∆PDR5 (AD) and wt-PDR5 (AD124567u^-^) were used as negative (0% Pdr5p expression) and positive (100% Pdr5p expression) controls, respectively. The 32 nucleotides upstream of the ATG start-codon for each construct are shown. *Sfi*I and *Pac*I restriction sites are underlined and the nucleotides that differ from the wild-type *PDR5* 5′ UTR are shown in bold type. The right hand column lists the MIC_FLC_ values for the strains. The MIC_FLC_ values for three independent transformants were measured and did not vary by more than one dilution. **C.** SDS-PAGE of plasma membrane proteins (30 μg) isolated from the strains listed in **B** including AD/PDR5 (this strain is AD with its wild-type *PDR5* locus restored). The black arrowhead indicates Pdr5p and the white arrowhead indicates the prominent plasma membrane proton pump protein Pma1p. SP = AD/SP-PDR5-URA3; S = AD/S-PDR5-URA3; P = AD/P-PDR5-URA3.

We have created an efficient system for the heterologous over-expression of fully functional membrane proteins in the uniquely modified *S. cerevisiae* host AD1-8u^-^ (AD) [14,15,17]. The list of successfully over-expressed membrane proteins includes important multidrug efflux pumps from a range of yeast species (e.g. *S. cerevisiae* Pdr5p, *C. albicans* Cdr1p and Cdr2p, *Candida glabrata* Cdr1p and Pdh1p, *Candida krusei* Abc1p and *Cryptococcus neoformans* Mdr1p [14,15]). The original expression system used the cloning vehicle pSK-PDR5-PPUS [15]. In an effort to improve the cloning efficiency for large genes such as fungal ATP-binding cassette (ABC) multidrug efflux pumps, pSK-PDR5-PPUS was modified to contain a range of conveniently positioned rare, 8 bp, restriction enzyme cloning sites (plasmid pABC1; Figure 1A). However, when *PDR5* was cloned either as an *Sfi*I/*Not*I or *Pac*I/*Not*I fragment into pABC1 (Figure 1A) there was an unexpectedly weak drug (fluconazole [FLC]) resistance phenotype conferred on host AD after integration of the transformation cassette into the genomic *PDR5* locus (compare MIC_FLC_ of AD/wt-PDR5 (400 mg/l) with AD/S-PDR5 and AD/SP-PDR5 (50 mg/l; Figure 1B)). This severely reduced drug resistance phenotype was reflected in dramatically reduced Pdr5p expression levels (Figure 1C).

Further tests showed that it was actually the *Sfi*I site with just 5 nucleotide changes to the 5′ UTR of *PDR5* that caused these ~8-fold reduced protein expression levels. This was confirmed by creating a set of *PDR5* over-expressing strains that had only the *Sfi*I, the *Sfi*I/*Pac*I, or the *Pac*I cloning sites in their 5′ UTRs: the rest of the *PDR5* locus was unchanged. Strain AD with the entire wt *PDR5* locus restored (AD/PDR5) was also created and served as a positive control. All AD derivative strains that had either a wt *PDR5* 5′ UTR, or strains that contained the *Pac*I site, had high Pdr5p expression levels (MIC_FLC_ = 400 mg/l; Table 1), but strains that contained the *Sfi*I site at position −18 relative to the ATG start codon had ~8-fold reduced MIC_FLC_ (50 mg/l; Table 1). This also clearly demonstrated that replacing the 3′ UTR of *PDR5* with the *PGK1* terminator followed by the *URA3* selection marker did not affect Pdr5p expression (Table 1). Based on these results we hypothesized that the unusual nature of the *Sfi*I cloning site (*Sfi*I = GGCCNNNNNGGCC) allowed the formation of a small, but stable, GC-rich, mRNA stem-loop 5′ proximal to the AUG start-codon that may have inhibited Pdr5p translation.

**Table 1:** **Effects of *PDR5 *5′ UTR and transcription terminator on Pdr5p expression in yeast strains AD and AD-sec6-4**

	**MIC**_**FLC**_**(mg/l)**	
***PDR5*5′ UTR**	**AD**	**AD sec6-4**
∆	0.6	0.6
WT	400	400
PacI	400	400
SfiI	50	50
SfiI/PacI	50	50
PacI	400	400
SfiI	50	nd
SfiI/PacI	50	50

^**+**^*∆PDR5* (top) = AD1-8u^-^. The strains underneath contain either the entire wt *PDR5* gene (WT) or were modified in their 5′ UTRs just upstream of the ATG start codon to create the restriction sites indicated in the neighbouring column. The strains in the bottom three rows are identical to the strains above except that the *PGK1ter* and *URA3* marker were placed between the ORF and the *PDR5* terminator.

nd = not determined, this strain was not constructed.

We therefore created plasmid pABC3, without the *Sfi*I site, which then enabled efficient cloning and maximal levels of expression of heterologous ORFs [14]. Clearly, as also demonstrated by Crook *et al.*, 2011 [18], MCSs are not just benign and convenient cloning tools but they can dramatically affect protein production in yeast, and sites with high GC content 5′ proximal to the ATG start codon should be used with caution. As the ability to reduce gene expression could be of use to us, we investigated the effect of the *Sfi*I site on Cdr1p expression.

### Inhibition of *PDR5* expression by the *Sfi*I cloning site is independent of its position, the host in which it is expressed, its sequence context, and growth conditions

Sec6p is part of the soluble eukaryotic exocyst complex that is required for the polarized transport of late post-Golgi secretory vesicles and their fusion with the plasma membrane. We created a temperature sensitive mutant in AD (AD/sec6-4) that enabled cells over-expressing Cdr1p to accumulate intact, late post-Golgi, secretory vesicles loaded with large amounts of mature Cdr1p after shifting the growth temperature for 2 h to the non-permissive temperature of 37°C [26]. Over-expression of different *PDR5* constructs in AD/sec6-4 gave identical FLC susceptibilities to those obtained for AD (Table 1), indicating that inhibition of expression of Pdr5p by the *Sfi*I-site was independent of the genetic background of the yeast host. Strains AD∆/P-CDR1-URA3 and AD/S-CDR1-URA3 were created using either pABC3 or pABC1 to over-express Cdr1p, the over-expression of which is often associated with multidrug resistance of the fungal pathogen *C. albicans*[27,28]. As with Pdr5p, the *Sfi*I-site caused a severe reduction (~4-fold) of the FLC resistance phenotype of AD/S-CDR1-URA3 compared to AD∆/P-CDR1-URA3 cells (Table 2). Interestingly, the FLC resistance of AD∆/P-CDR1-URA3 (MIC_FLC_ = 200 mg/l) was also ~6-times higher than AD1002 (MIC_FLC_ = 30 mg/l) - the original *CDR1* over-expressing strain that was created using pSK-PDR5-PPUS [15]. AD1002 contained seven hexamer cloning sites (5′ *Hin*dIII, *Eco*RV, *Eco*RI, *Pst*I, *Sma*I, *Bam*HI, *Spe*I 3′) 5′ proximal to the *CDR1* ATG start codon [15]. Expressing two other ORFs (*C. albicans* multidrug efflux pump *CDR2 A* and *B* alleles [29]) as either *Pac*I or *Sfi*I fragments gave similar results. The *Sfi*I-site reduced the expression of *CDR2A* ~6-fold and the expression of *CDR2B* ~8-fold (Table 2). These results clearly demonstrated that the degree of inhibition (<4-8-fold) of expression by the *Sfi*I-site was also largely independent of the adjacent ORF sequence. The inhibition of Pdr5p, Cdr1Ap and Cdr2Ap expression by the *Sfi*I-site was also unaffected by changing the pH of the growth medium, replacing glucose with non-fermentable glycerol as a carbon source or replacing the synthetic CSM with the complex YPD growth medium (Table 3). And creating AD∆/SfiI(−4)-CDR1 with the *Sfi*I-site positioned at −4 instead of −18 (AD/S-CDR1-URA3) relative to the ATG start-codon showed that inhibition of Cdr1p expression by the *Sfi*I-site was also independent of its position in the 5′ UTR near the ATG start codon (Table 2).

**Table 2:** **Effects of *PDR5 *5′ UTR on Cdr1p and Cdr2p expression**

**AD/**^**+**^	***PDR5*5′ UTR**^**++**^	**MIC**_**FLC**_**(mg/l)**
*CDR1A*	PacI	200
	SfiI	50
	SfiI(−4)	50
*CDR2A*^+++^	PacI	120
	SfiI	20
*CDR2B*^+++^	PacI	240
	SfiI	30

^+^ AD strains containing the indicated ORFs followed by the *PGK1ter* and *URA3* marker.

^++^ Nucleotide changes to the *PDR5* 5′ UTR just upstream of the ATG start codon (SfiI(−4) = CDR1-construct #1).

^+++^ The two alleles of *CDR2* from *C. albicans* ATCC 10261.

**Table 3:** Effects of growth conditions on Pdr5p, Cdr1p, and Cdr2p expression

		**MIC**_**FLC**_**(mg/l)**			
		**CSM**			**YPD**
AD/^*^	loop^+^	pH 7.0	pH 5.6	glycerol^++^	
*∆PDR5*	-	0.8	3.1	3.1	3.1
*PDR5*	wt	600	600	600	600
	SfiI	75	75	75	37.5
*CDR1A*	PacI	300	600	600	300
	SfiI-9	37.5	75	75	75
*CDR2A*	PacI	100	100	100	200
	SfiI	12.5	12.5	25	12.5

^*^∆PDR5 = AD; *PDR5*: wt = AD/PDR5 and S = AD/S-PDR5; *CDR1A*: *Pac*I = AD∆/P-CDR1-URA3 and *Sfi*I-9 = AD∆/construct-9-CDR1; *CDR2A*: *Pac*I = AD/P-CDR2A-URA3 and *Sfi*I = AD/S-CDR2A-URA3 (Table 5).

^**+**^ Nucleotide changes to the *PDR5* 5′ UTR just upstream of the ATG start codon.

^**++**^ Glycerol instead of glucose in the CSM.

### The *Sfi*I-site in the 5′ UTR near the ATG start codon inhibits translation and increases steady-state mRNA levels

**Figure 2:**
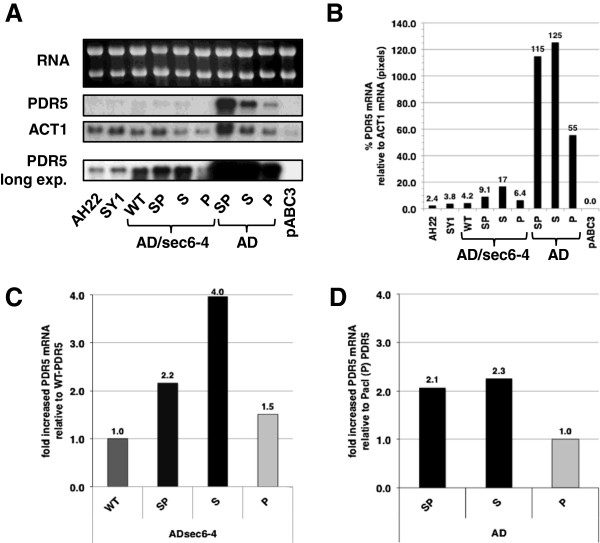
**The *Sfi*I-site in the 5′ UTR near the AUG start codon increases the levels of *PDR5 *mRNA.** Northern blot analysis was performed on late-logarithmically grown AD and AD/sec6-4 cells that expressed *PDR5* with differently modified 5′ UTRs (WT = unmodified; strains labeled SP, S or P contained the SfiI/PacI, the SfiI or the PacI site, respectively). AD strains contained the *PGK1* terminator and the *URA3* marker at the 3′ end of *PDR5* while AD/sec6-4 strains contained the 3′ UTR of *PDR5*. Three control strains were included: two wild-type *PDR5* expressing strains (AH22 and SY1) and AD/pABC3 as the negative *(∆PDR5*) control. **A** Upper panel - 10 μg total RNA extracts separated on a 1.2% denaturing agarose gel and stained with EtBr (top), lower panels - autoradiographs of blots probed with *PDR5* and *ACT1*. The band intensities for the top two *PDR5* and *ACT1* panels can be directly compared as they experienced the same treatment (*PDR5* and *ACT1* probes were combined for the hybridization with the Northern blot) while the autoradiograph at the bottom was overexposed so that *PDR5* bands of weaker intensities could be measured accurately. **B**, **C**, and **D** show the intensities of bands in panel A quantified with the ImageJ software program [30]. **B** shows the expression of *PDR5* relative to the expression of the housekeeping gene *ACT1* that was used as an internal standard (the intensities for *ACT1* in AD/SP-PDR5 were ~10-times higher than in AD/pABC3 while *ACT1* varied no more than +/− 50% in the remaining samples). **C** and **D** show the -fold differences in normalized *PDR5* mRNA levels relative to AD/sec6-4/PDR5 **(C)** and AD/P-PDR5 **(D)**, respectively (the results for the *Sfi*I-site containing strains are shown with black bars, for wt-PDR5 with dark grey and *Pac*I-site containing strains with light grey bars). The numbers above individual bars in **B**, **C**, and **D** give the actual values represented by the bars.

To ascertain whether the reduced expression of Pdr5p in strains containing the *Sfi*I-site was due to reduced *PDR5* mRNA levels or whether the *Sfi*I-site inhibited translation of Pdr5p, Northern blot analysis of late-logarithmic cells (grown in CSM and harvested at OD_600_ = 5) was performed on AD and AD/sec6-4 cells over-expressing different *PDR5* mRNA-constructs (Figure 2). Two wild-type control strains (AH22 and SY1) and the negative (*∆PDR5*) control strain AD/pABC3 were also included, and the housekeeping gene *ACT1* was used as an internal standard. *PDR5* mRNA levels, normalized for *ACT1*, were very low (~2-4%) in AH22 and SY1 (without Pdr1-3p) and, as expected, no *PDR5* mRNA was detectable for AD/pABC3 (Figure 2A and B). Interestingly though, normalized *PDR5* mRNA levels were ~10-times higher in AD (55-125%) than in AD/sec6-4 (4.2-17%; Figure 2B), although expression of Pdr5p was the same in either genetic background (Table 1). Indeed the *PDR5* mRNA levels of the wt- and *Pac*I-site containing constructs in AD/sec6-4 were very similar to those in SY1 and AH22 (Figure 2B). However, despite these significant differences in mRNA levels of *PDR5* in AD/sec6-4 and AD which could be due to the different transcription terminators, the levels in all *Sfi*I-site containing constructs were reproducibly 2-4-times higher than in their respective *Pac*I (Figure 2C and D) or WT (Figure 2C) constructs, possibly due to an mRNA-stabilizing effect. This effect was independent of the amount of mRNA present in the cell: *PDR5* mRNA levels were ~3-times higher for the *Sfi*I-site containing constructs when cells (AD/sec6-4/PDR5, AD/sec6-4/P-PDR5 and AD/sec6-4/S-PDR5) were harvested either at early-log (2.5-times higher), late-log (3.4-times higher), or stationary growth phase (2.9-times higher; Additional file 1: Figure S1E), even though the *PDR5* mRNA levels were ~10-times higher in early-log than late-log or stationary phase cells (Additional file 1: Figure S1C).

These results clearly demonstrated that the *Sfi*I-site 5′ proximal to the AUG start codon inhibited translation of Pdr5p in the presence of a ~3-fold increase in *PDR5* mRNA levels that was independent of: i) steady-state mRNA levels; and ii) the growth stage of cells.

### The *Sfi*I mRNA stem-loop provides a strong physical barrier for the yeast translation initiation machinery

To analyze the inhibitory effect of the *Sfi*I mRNA stem-loop on the expression of Cdr1p in more detail, and to ascertain whether we could predictably tune expression by modifying mRNA stem-loop structures positioned at −4 relative to the AUG start codon, we created Cdr1p-expressing yeast strains with 17 different, systematically modified, GC-rich mRNA stem-loops near the AUG start codon. A detailed description of the strategy employed to create these different mRNA species is given in the Materials and Methods section, and a schematic illustration can be found in Additional file 2: Figure S2.

In order to establish whether the *Sfi*I mRNA stem-loop *per se* or a specific *Sfi*I mRNA stem-loop-recognizing protein caused the inhibition of translation, we systematically modified the size of the stem and the size of the loop of the hypothetical *Sfi*I mRNA stem-loop in front of the AUG start codon and quantified the effects. mRNA secondary structure predictions and calculation of their thermodynamic stabilities (Table 4) were determined with the Mfold web server [31]. Increasing the size of the hypothetical *Sfi*I loop from 5 nucleotides (GCUCG, construct 1) to 8 (AAGCUCGA, construct 6) or decreasing it to 2 nucleotides (AA, construct 7) caused only moderate (~2-fold) increases in their respective MIC_FLC_ values (Table 4). Only the complete elimination of the loop (construct 8) restored wt-Cdr1p expression levels (Table 4). Slightly increasing the thermodynamic stability of the *Sfi*I stem-loop of construct 1 by replacing an AU-pair with a GC-pair in construct 2 (∆G = −9.8 vs. -8.6 kcal/mol) caused a further ~4-fold reduction of Cdr1p expression (Table 4) while reducing the size of the stem from five (construct 2) to two GC-pairs (construct 5) eliminated any possible secondary structure and resulted in wt-Cdr1p expression levels (Table 4). Construct 4, however, with a stem of only 3 GC-pairs (∆G = −4.4 kcal/mol), was still able to inhibit Cdr1p expression by ~50% (Table 4).

**Table 4:** **Effects of modifying the core *Sfi *I stem-loop sequence (GGCCGCTCGGGCC; modifying the size of the stem and the loop) at position −4 to the ATG start codon on the expression of Cdr1p**

**construct #**	**5′ UTR**^**+**^		**stem-loop type**	**MIC**_**FLC**_^**++**^**(mg/l)**	**-∆G**^**+++**^**(kcal/mol)**
			**loop +**		
**6**	TCCGCTCGAGGCC***AA*GCTCG*A***GGCCTAAAATG		**8**	**100**	**8.6**
**7**	TCCGCTCGTTCGAAAGGCC***AA***GGCCTAAAATG		**2**	**100**	**6.6**
**8**	TCCGCTCGTTCGAAAGAGGCCGGCCTAAAATG		**0**	**200**	**2.8**
**1**	TCCGCTCGTTCAGGCC**GCTCG**GGCCTAAAATG		**5**	**50**	**8.6**
			**stem +**		
**5**	TCCGCTCGTTCGA**TT**CCGCTCGGGCCAAAATG		**−2**	**200**	**−0.2**
**4**	TCCGCTCGTTCGAGGCCGCTCGGGC**G**AAAATG		**−1**	**100**	**4.4**
**9**	TCCGCTCGAAAAGGCCGCTCGGGCCAAAAATG		**-**	**25**	**7.9**
**2**	TCCGCTCGTTC**C**GGCCGCTCGGGCC**G**AAAATG		**C**	**12.5**	**9.8**
**11**	TCCGCTCGAAA**C**GGCCGCTCGGGCC**G**AAAATG		**C**	**12.5**	**10.0**
**14**	TCCGCTAAA**GC**GGCCGCTCGGGCC**GC**AAAATG		**GC**	**3.2**	**13.7**
**15**	TCCGAAA**CGC**GGCCGCTCGGGCC**GCG**AAAATG		**CGC**	**1.6**	**15.8**
1*	TCCGCTCGTTC**A**GGCCGCTCGGGCC**T**AAAATG		**A**	**50**	**8.6**
10*	TCCGCTCGAAA**T**GGCCGCTCGGGCC**A**AAAATG		**T**	**6.3**	**8.7**
13*	TCCGCTAAA**TA**GGCCGCTCGGGCC**TA**AAAATG		**TA**	**25**	**10.0**
18*	TCCGAAA**TTA**GGCCGCTCGGGCC**TAA**AAAATG		**TTA**	**12.5**	**10.9**
12*	TCCGCTAAA**GA**GGCCGCTCGGGCC**TC**AAAATG		**GA**	**6.3**	**12.4**
17*	TCCGAAA**TGA**GGCCGCTCGGGCC**TCA**AAAATG		**TGA**	**3.2**	**13.2**
16*	TCCGAAA**CGA**GGCCGCTCGGGCC**TCG**AAAATG		**CGA**	**6.3**	**14.5**

^**+**^ All stem-loop sequences are underlined. Loop nucleotides of the *Sfi*I stem-loop constructs with different size loops (top) are highlighted in bold type and nucleotide changes made to modify the size of the stem (bottom) of the core *Sfi*I stem-loop are also highlighted in bold type letters. Numbers at the top panel indicate the size of the loop; a – sign followed by a number indicates by how many nucleotide pairs the core *Sfi*I stem-loop stem was reduced and letters indicate the nucleotide sequence of the left arm of the nucleotide pairs with which the core *Sfi*I stem-loop had been increased.

^**++**^ MIC_FLC_ values represent the MIC_FLC_ values of three independent transformants and they were practically identical for all constructs tested.

^**+++**^ ∆G values calculated for individual *Sfi*I stem-loops (underlined sequences).

*SfiI stem-loop constructs of different size stems containing mixed AU- and GC-pairs.

These results were consistent with an *Sfi*I site at −4 that forms a GC-rich mRNA stem-loop which provides a strong physical barrier for the yeast ribosomal AUG start codon scanning machinery.

### FLC resistance levels are an accurate measure for the amounts of Cdr1p expressed

**Figure 3:**
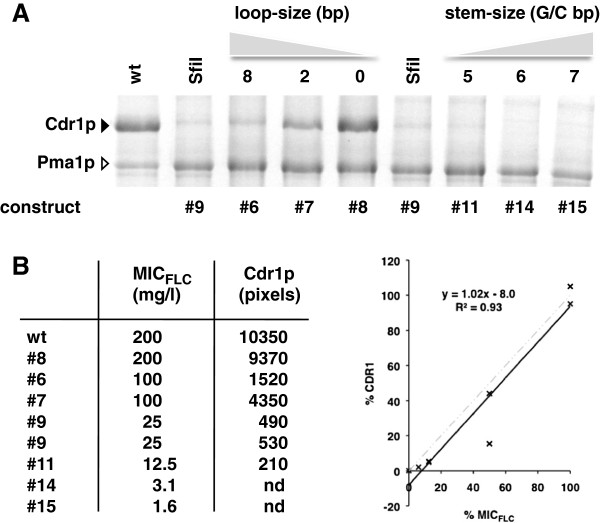
**Drug resistance levels (MIC**_**FLC**_**) of Cdr1p-expressing strains are directly proportional to the amount of Cdr1p expressed. A.** SDS-PAGE of plasma membrane proteins (30 μg) isolated from AD∆ strains containing different *Sfi*I stem-loop constructs. The black arrowhead indicates Cdr1p and the white arrowhead indicates the prominent plasma membrane proton pump protein Pma1p. wt = AD∆/P-CDR1-URA3; SfiI = AD∆/construct9-CDR1; lanes labeled 8, 2 and 0 represent Cdr1p expressing strains with decreasing loop-size of 8 nucleotides (AD∆/construct6-CDR1), 2 nucleotides (AD∆/construct7-CDR1) or no loop at all (AD∆/construct8-CDR1); lanes labeled 5, 6 and 7 represent strains with increasing stem-size of 5 GC-pairs (AD∆/construct11-CDR1), 6 GC-pairs (AD∆/construct14-CDR1) and 7 GC-pairs (AD∆/construct15-CDR1). **B.** The MIC_FLC_ values for each construct correlated well with the amounts of Cdr1p expressed (measured as pixels using the ImageJ software [30]). %CDR1 (Y-axis) and %MIC_FLC_ (X-axis) are the expression levels and MIC_FLC_ relative to wt Cdr1p. To the right is a graphical illustration of this correlation (constructs #14 and #15 were excluded from the graph because their Cdr1p expression was below the detection limit but the MIC_FLC_ = 0.5 of the negative control strain AD (no Cdr1p) was included), and the dashed grey line shows the theoretical trend line expected for a direct linear correlation between MIC_FLC_ values and the amounts of Cdr1p expressed.

To confirm that FLC resistance levels can be used as an accurate measure for the amount of Cdr1p expressed, we isolated plasma membranes from a representative set of strains and compared their MIC_FLC_ values with the amount of Cdr1p expressed (Figure 3). A plot of the amount of Cdr1p expressed and the measured FLC resistance level for seven of these constructs (including the MIC_FLC_ value for the negative control strain AD; MIC_FLC_ = 0.5 mg/l) showed that there was a strong linear correlation (R^2^ = 0.93; Figure 3B; construct 6 (4 GC-pairs stem plus 8 bp loop; MIC = 100 mg/l) appeared to be an outlier due to the 50% margin of error intrinsic to the way MIC values were measured). This confirmed that the MIC_FLC_ values of different Cdr1p expressing strains could be used as a simple measure for Cdr1p expression levels. The data also seemed to suggest that all Cdr1p molecules were fully functional and contributed equally/additively to FLC transport even when expressed at levels as high as ~30% of total plasma membrane protein.

### Cdr1p expression levels reduce exponentially with the number of GC-pairs in mRNA stem-loop stems containing only GC-pairs

**Figure 4:**
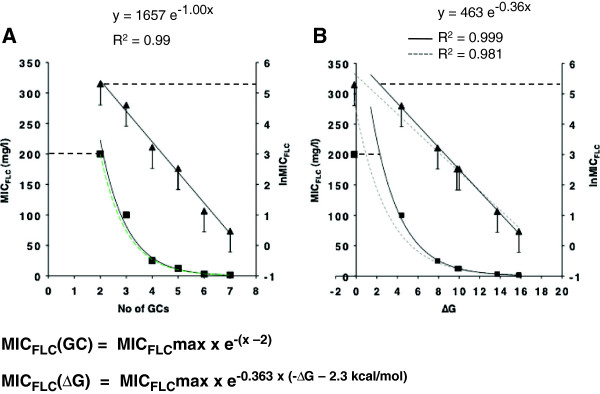
**Cdr1p expression levels decrease exponentially with an increase in the number of GC-pairs in stems containing only GC-pairs 5′ proximal to the AUG start-codon. A.** The relationship between MIC_FLC_ values of Cdr1p-expressing strains and the number of GC-pairs in stems containing only GC-pairs. Black squares represent MIC_FLC_ values and black triangles represent the lnMIC_FLC_ values plotted against the number of GC-pairs in stem-loop constructs. The two black lines represent the trend lines for the best fit of these two data sets (‘error’ bars indicate the possible range of MIC_FLC_ values of individual constructs; see text for further details). The horizontal black dashed lines mark the MIC_FLC_max values of wild type Cdr1p-expressing cells. The green dashed trend line is the trend line that was created with the calculated MIC_FLC_ values for stem-loop constructs containing only GC-pairs using the formula shown underneath. **B.** The relationship between MIC_FLC_ values of Cdr1p-expressing strains and the calculated ∆G values for stems containing only GC-pairs. The same symbols as in **A** were used. The dashed grey lines are the trend lines for the best fit including the data point for the construct containing 2 GC-pairs, while the two black lines represent the trend lines for the best fit of the data sets excluding that data point. The formula that can predict the MIC_FLC_ values for stem-loop constructs containing only GC-pairs using their calculated ∆G values is shown underneath. All MIC_FLC_ values are the values for three independent transformants and did not vary by more than one dilution.

The step-wise increase in GC-pairs of the ‘core’ *Sfi*I mRNA stem-loop stem (construct 9; Table 4) led to a remarkably consistent decrease in Cdr1p expression: constructs 2 and 11 (+1 GC-), 14 (+2 GC-) and 15 (+3 GC-pairs) each expressed ~50-75% less Cdr1p than the previous construct (Figure 4A and Table 4).

Surprisingly, the MIC_FLC_ values decreased exponentially with the number of GC-pairs in mRNA stem-loop constructs containing only GC-pairs (Figure 4A). Even the two smallest 2 and 3 GC-pairs stem constructs 5 and 4 fit well (R^2^ = 0.99) onto the black exponential trend lines (Figure 4A). This was even more remarkable given the 50% margin of error that was intrinsic to the way MIC_FLC_ values were determined (see ‘error’ bars in Figure 4).

There was also a clear exponential relationship between the thermodynamic stabilities of these constructs and their MIC_FLC_ values (R^2^ = 0.98; dashed grey trend lines in Figure 4B). However, in this case the variance decreased when the data point for the smallest, 2 GC-pairs, stem-loop construct 5 was excluded (R^2^ = 0.999; black trend lines in Figure 4B).

The exponential dependency of Cdr1p expression levels (MIC_FLC_) for cells with mRNA stem-loops containing only GC-pairs with stems ≥3 GC-pairs on either: i) the number of GC-pairs (MIC_FLC_(GC)); or ii) the thermodynamic stabilities (MIC_FLC_(∆G)) of these constructs could be expressed as the formulae shown at the bottom of Figure 4 (x is the number of GC-pairs). The dashed green trend line in Figure 4A represents the calculated trend line for these constructs (assuming a MIC_FLC_max = 200 mg/l). It matched the experimentally determined results (black trend line) exceptionally well.

We conclude that even an mRNA stem-loop of 3 GC-pairs is biologically active and able to provide a relatively strong physical barrier for the yeast 43S PIC.

### *Sfi*I mRNA stem-loop stems of mixed AU/GC-pair stems

Eliminating the single AU-pair of the original *Sfi*I stem-loop construct 1 to form construct 9 caused an unexpected ~2-fold reduction of Cdr1p expression (Table 4). Adding 1, 2, or 3 additional AU-pairs to construct 9 also led to unpredictable results: one extra AU-pair (construct 10) caused a ~4-fold reduction in expression rather than the 2-fold increase observed for construct 1 (construct 10 had the same number of AU/GC-pairs as construct 1 but the additional AU pair was arranged in an inverted fashion and the three nucleotides 5′ proximal to the stem-loop of construct 1 (TTC) were replaced with AAA in construct 10; Table 4), two extra AU-pairs (construct 13) had no apparent effect, while three additional AU-pairs (construct 18) gave only a ~2-fold reduction of Cdr1p expression compared with construct 9 (Table 4). Clearly, the presence of additional AU-pairs in GC-rich stem-loops (constructs 1, 10, 12, 13, 16, 17 and 18; Table 4) led to less predictable Cdr1p expression levels and their effects appeared to be dependent on the surrounding mRNA sequence unlike mRNA stem-loops containing only GC-pairs whose inhibitory effects remained unaffected by the surrounding mRNA sequence (e.g. constructs 2 and 11 had identical MIC_FLC_ values although they contained the same nucleotide variations as constructs 1 and 10; Table 4).

### A practical application of GC-rich mRNA stem-loops for high-throughput drug screening

**Figure 5:**
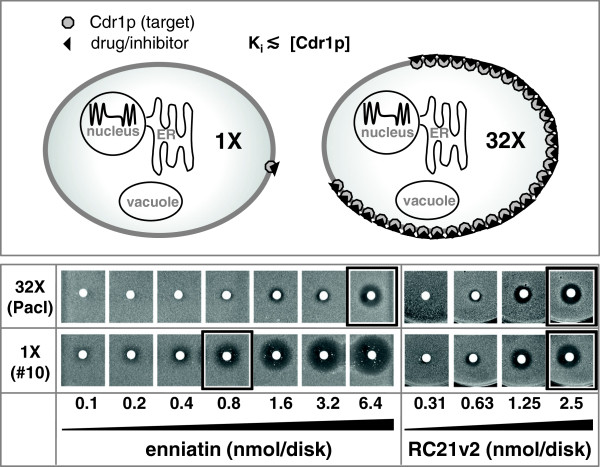
**Demonstration of Cdr1p target concentration-dependent chemosensitization to enniatin and RC21v2.** The top panel illustrates the theoretical assumption that 32 times more of a high-affinity Cdr1p inhibitor (K_i_ ≪ [Cdr1p]) is required to inhibit a strain that expresses 32 times more Cdr1p. Yeast strains AD∆/P-CDR1-URA3 (32X, top row) and AD∆/construct10-CDR1 (1X, bottom row) were used to test this theory. CSM agar plates contained [FLC] = ¼ MIC_FLC_ of these two test strains (Table 4). Filter disks containing 0.1 - 6.3 nmol of enniatin or 0.3 - 2.5 nmol of RC21v2 (applied at two-fold increasing amounts) were placed onto these plates after they had been seeded with yeast cells and incubated for two days at 30°C. The size of the growth inhibitory zones was used as a visual indicator for the level of Cdr1p inhibition. While ~10 times more enniatin was needed to inhibit wt-Cdr1p expressing cells (boxed disks) almost identical amounts of the Cdr1p inhibitor RC21v2 were required to inhibit the two test strains (boxed disks).

The ability to stably express heterologous genes at different levels has exciting applications in drug screening. Yeast provides a good platform for high-throughput drug screening due to its genetic tractability, its fast growth rate, its scalability, and its ability to grow on relatively inexpensive growth media. Screening large compound libraries can yield high numbers of false positive hits. One way to reduce the number of false positive hits would be to perform secondary screens with two additional strains: i) a negative control strain that does not express the target to identify false positive hits caused by general toxicity of the compound; and ii) a strain that expresses significantly lower amounts of the target protein (e.g. ~32 times less; Figure 5; top panel) to increase the success rate for identifying positive hits that inhibit the target protein with highest efficiency (i.e. preferred hits). Growth inhibition of a strain that expresses ~32 times less of an essential target would (in theory) require ~32 times less of a highly effective compound (compound has high affinity to the target) but similar amounts of a less effective (low-affinity) compound. This is because high-affinity inhibitors (i.e. Ki ≪ target concentration [E]; Figure 5), unlike low affinity inhibitors (Ki ≧ [E]), do not follow the typical Michaelis-Menten kinetics.

We demonstrated how such a screen may be used to distinguish between a strong, target-specific, inhibitor of efflux pump Cdr1p, enniatin [32], and RC21v2 [33], a weaker Cdr1p-specific D-octapeptide inhibitor (Figure 5). CSM agar plates contained FLC at concentrations of ¼ the MIC_FLC_ of the test strains so that each strain was able to grow and cells accumulated similar amounts of FLC. The assumption that the test strains accumulate similar amounts of intracellular FLC is based on the fact that the FLC drug target Erg11p is located inside the cell in the endoplasmic reticulum and that, while the test strains differ in the amounts of Cdr1p expressed, they express the same, or very similar, amounts of Erg11p and therefore require the same, or very similar, intracellular concentrations of FLC to inhibit its essential function. Conducting the experiment in this way ensured that Cdr1p inhibition was directly dependent on the amount of inhibitor used. Two-fold increasing amounts of enniatin or RC21v2 were put on filter disks and the disks were placed onto plates seeded with a lawn of either of the two strains (Figure 5). After incubating the plates at 30°C for two days growth inhibitory zones appeared, the sizes of which were used as an indication of the level of Cdr1p inhibition. wt-*CDR1* expressing cells required ~8-16 times more enniatin than *CDR1*-construct 10 expressing cells whereas both Cdr1p-expressing strains required similar amounts of the weaker inhibitor RC21v2 to inhibit cell growth to the same degree (Figure 5).

## Discussion

Our host strain AD∆ is deleted in seven ABC transporters [13] and therefore exquisitely sensitive to many xenobiotics [14,15]. The overexpression of Cdr1p led to a ~400-fold increase in FLC resistance. We exploited this large dynamic range of FLC susceptibilities as a very sensitive and robust tool to analyze the effects of varying mRNA stem-loops 5′ proximal of the AUG start-codon on the efficiency of Cdr1p translation in yeast.

A number of studies have shown that small, GC-rich, mRNA stem-loops placed into 5′ UTRs of yeast genes have strong inhibitory effects on their expression levels [19-24]. This effect was exhibited at the level of translation (mRNA levels were mostly unaffected and varied no more than 2–4 fold [19,22,24]) and was largely independent of gene context and the promoter used. The inhibitory effects of individual mRNA stem-loops were comparable to some of our Cdr1p stem-loop constructs (Table 4): e.g. i) a −10.5 kcal/mol mRNA stem-loop (*GAATTCCC*ATCTT*GGGAATTC*; stem nucleotides are in italics) positioned 21 nt upstream of the AUG start-codon of the *GCN4-lacZ* reporter plasmid reduced the β-galactosidase activity to 13% [20]; and ii) a −8.5 kcal/mol mRNA stem-loop (*TGAATTCG*TTAA*CGAATTCA*) right next to the AUG start codon of the *CYC1* gene (this construct was integrated into the *CYC1* locus) reduced iso-1-cytochrome c expression to 10% [19]. Most other mRNA stem-loops tested were of higher stabilities (<−20 kcal/mol) and inhibited reporter gene expression (e.g. endogenous *CYC1* and *HIS4* genes or the plasmid-encoded chloramphenicol acetyl transferase (*cat*) reporter) to <1% or completely undetectable levels causing histidine auxotrophy for some *HIS4* constructs [19,21,24].

**Figure 6:**
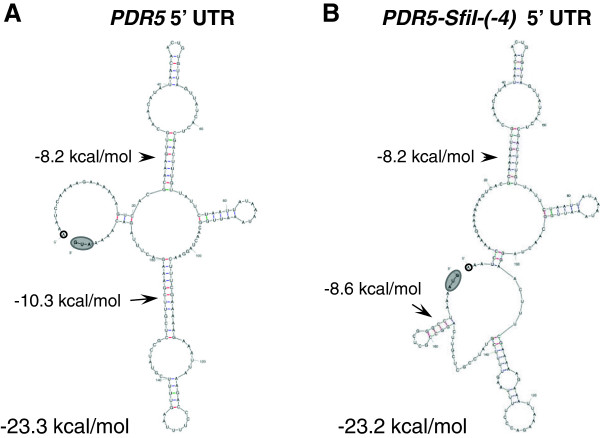
**mRNA secondary structures predicted for the 5′ UTRs of wild-type *PDR5 *and *PDR5 *containing the originally created *Sfi *I stem-loop at −4 (construct #1).** The most representative mRNA secondary structures predicted for the entire 5′ UTRs of wild-type *PDR5* (three transcription start sites were determined at −171, -174 and −175 [35]) and wild-type *PDR5* that has been modified to contain the *Sfi*I site at −4 (construct #1) are shown in **A** and **B**, respectively. Arrows with numbers (kcal/mol) indicate secondary structures of significant stability. 5′ ends are highlighted with black circles and the AUG start-codons are highlighted as grey ovals. The calculated thermodynamic stabilities for the entire 5′ UTR of each predicted secondary structure is shown at the bottom of each structure.

However, our studies demonstrate for the first time that small GC-rich mRNA stem-loops placed 5′ proximal to the AUG start-codon can be used as an efficient tool to ‘predictably’ down-regulate protein expression levels in yeast whereas the degree of inhibition of mRNA stem-loops of mixed G/C and A/U pairs was less predictable. Figure 6 shows representative secondary structures predicted for the wild-type and the *Sfi*I-site containing 5′ UTRs of *PDR5*. All 34 possible secondary structures for the *Sfi*I-containing construct predicted the presence of the −8.6 kcal/mol GC-rich mRNA stem-loop (Figure 6B) but the structures for the rest of the molecule varied significantly (data not shown). This −8.6 kcal/mol GC-rich *Sfi*I mRNA stem-loop was also predicted for all possible secondary structures that included the 5′ part of *CDR1* (data not shown), indicating that the surrounding mRNA sequence has little effect on the possible formation of small, GC-rich, mRNA stem-loops. We experimentally verified sequence context independence of the inhibitory effects of this *Sfi*I mRNA stem-loop by expressing four different ORFs (*PDR5*, *CDR1*, *CDR2A* or *CDR2B*) and using two different terminators (*PGK1* or *PDR5* terminator) with similar results. The inhibitory effects of the *Sfi*I mRNA stem-loop were also independent of the host in which they were expressed or any changes to the growth medium. Interestingly, Northern blot analysis revealed that the *Sfi*I mRNA stem-loop increased (~3-fold) the steady-state mRNA levels of *PDR5*. This increase was observed in different strain backgrounds and in cells harvested at different growth stages. Thus, it seemed that the GC-rich mRNA stem-loop had increased the stability (i.e. half-life) of the mRNA. Sagliocco *et al.*, 1994, made similar observations for a *cat* reporter transcript that contained a strong (∆G = −23.2 kcal/mol) GC-rich mRNA stem-loop (the stem had 8 consecutive GC-pairs) placed in its 5′ UTR which inhibited its translation by ~99% but increased its mRNA half-life ~3-fold [34]. However, further investigations revealed no obvious correlation between the translatability of different stem-loop constructs and their half-life [34].

**Figure 7:**
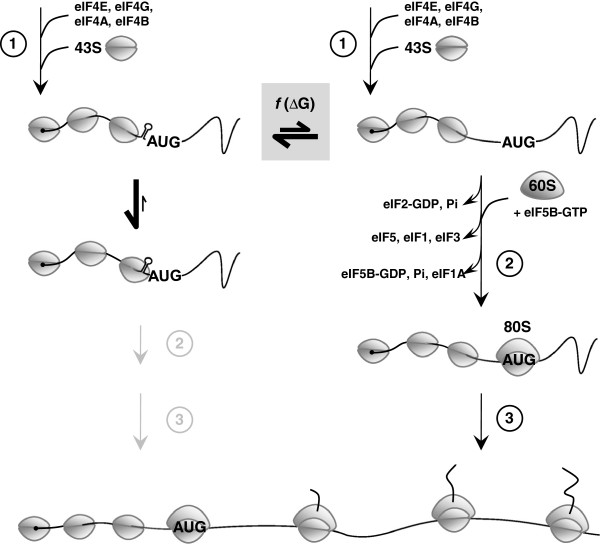
**Effects of small, ‘local’, GC-rich mRNA stem-loops on the yeast 43S PIC.** Protein translation in eukaryotes starts (**1**) with the binding of the eukaryotic initiation factor 4F (eIF4F = eIF4E and eIF4G) to the 5′ cap (5′ m^7^G; black dot) of the mature mRNA. eIF4A together with eIF4B are thought to unwind secondary structure in an ATP-dependent fashion close to the 5′ cap to allow access for the 43S PIC (small grey ovals). The 43S PIC consists of the 40S ribosomal subunit to which eIF1, 1A, 2 (bound to GTP), 3 and 5 and tRNA^Met^ are bound. After attachment of the 43S PIC next to the 5′ cap region AUG start-codon scanning proceeds. Recognition of the AUG start-codon (**2**) induces GTP hydrolysis and the release of eIF2, GDP, and Pi, which is followed by the eIF5B-GTP catalyzed joining of the 60S subunit (large grey oval) and the displacement of eIF5, eIF1 and eIF3 followed by hydrolysis of eIF5B-GTP and the release of eIF5B-GDP and eIF1A leading to the production of an 80S initiation complex competent for elongation (**3**) [36,37]. Small, ‘local’, GC-rich mRNA stem-loops 5′ proximal to the AUG start codon inhibit AUG start-codon scanning of the yeast 43S PIC by providing a physical barrier to the advancing complex and possibly get trapped inside the complex (center left; [36,40]). The ratio between mRNA molecules that contain a GC-rich mRNA stem-loop (left side) in front of the advancing 43S PIC and those that don’t (right side) may be a function of the thermodynamic stability of stem-loops and determine the amount of protein that can be translated leading to a biphasic polysome distribution (the majority of mRNA molecules bound to 43S PIC (center left) and a small pool of mRNA that is inversely proportional to the stability of the stem-loop and heavily loaded with actively translating 80S ribosomes (bottom)).

The vast amount of literature on translation initiation in yeast and higher eukaryotes (a schematic illustration is given in Figure 7 and reviews can be found in [36-38]) combined with our own observations reveal important insights into the possible molecular mechanism of inhibition of translation by GC-rich mRNA stem-loops in yeast. A first important clue derives from studies by Sagliocco *et al.*, 1993, who clearly demonstrated that any mRNA reporter that carried a strong 5′-secondary structure had a biphasic polysome distribution one mRNA pool that was actively translated and heavily loaded with ribosomes while the majority of mRNA molecules had only the 43S PIC bound [23]. And secondly, studies have shown that the 43S PIC is extremely tightly bound to the mRNA *in vivo*[39]. This together with our own observations (i.e. inhibition of AUG start codon scanning by small stem-loops is directly proportional to the predicted thermodynamic stability (*f*(∆G); grey boxed-in area) of stem-loop stems consisting of GC-pairs only) is strongly suggestive of the following model of AUG start codon scanning of the yeast 43S PIC. There are two types of transcripts (shown at the top of Figure 7) that are in equilibrium with each other, those that have the GC-rich mRNA stem-loop formed in front of the advancing 43S PIC (left) and those that don’t (right). GC-rich stem-loop containing mRNA species get stuck in the advancing 43S PIC (i.e. the yeast 43S PIC cannot resolve stems >2 GC-pairs; center left of Figure 7) leaving only the small remaining pool of mRNA without a GC-rich stem-loop to be efficiently translated (once the first 43S PIC has successfully scanned through the entire 5′ UTR the mRNA remains unfolded [23]; right side of Figure 7), leading to a biphasic polysome distribution and inhibition of translation that is inversely proportional to the thermodynamic stability of the stem-loop. This model is further supported by recent *in vitro* studies with a reconstituted mammalian translation system where the authors have shown that small GC-rich mRNA stem-loops get trapped inside the 43S PIC and require a dedicated DHX29 RNA helicase to be resolved [40]. Interestingly, yeast does not appear to have a dedicated DHX29 homolog [40] which could explain why it is so sensitive to GC-rich mRNA stem-loops artificially introduced into its 5′ UTRs and why their rather short 5′ UTRs (<134 nucleotides [41]) are so AU rich [41,42]. Clearly, further experimental evidence is required to unravel whether GC-rich mRNA stem-loops do indeed get stuck inside the 43S PIC and create a ‘stalled’ complex from which it is difficult for the yeast 43S PIC to escape (center left; Figure 7), or whether another less obvious mechanism is at work that could explain our findings. But whatever the mechanism, it is clear that the yeast 43S PIC has a very limited ability to unwind ‘local’ (i.e. complementary stem nucleotide-pairs separated by loops of no more than ~10 nucleotides) GC-rich mRNA stem-loops and their degree of inhibition of translation is determined solely by their thermodynamic stability and independent of sequence context and the promoter used.

## Conclusions

We successfully exploited the intrinsic nature of small GC-rich mRNA stem-loop modules 5′ proximal to the AUG start-codon of yeast genes to stably and predictably tune gene dosage from a single promoter without the need for inducers. This discovery, and the general lack of well-characterized promoters for gene expression in yeast, makes GC-rich mRNA stem-loop modules an important tool for regulating protein expression in yeast. They could be of value for i) the titration of minimal expression levels required for essential genes; ii) the elucidation of gene function; or iii) the determination of the precise impact of the gene dosage on a desired phenotype [11]. They could help identify the rate-limiting step and optimize the expression levels for genes in novel metabolic pathways by modifying the expression modules for each gene. Also they could be used to down-regulate expression levels of essential genes of competing endogenous biological pathways, which can lead to dramatically reduced levels of a target metabolite. One example would be the successful synthesis of artemisinic acid, precursor of the antimalarial artemisinin, in yeast that required down-regulation of the essential gene *ERG9*[8,43]. Another application of the GC-rich mRNA stem-loop modules is the optimization of heterologous membrane protein expression in yeast as, often, high expression levels can lead to their misfolding and/or mislocalization [44,45]. As AUG start-codon scanning is a universal eukaryotic feature it is possible that this strategy can be applied in many other eukaryotic hosts as well [25].

## Materials and methods

### Strains and culture conditions

*S. cerevisiae* strains used in this study are listed in Table 5 and are based on AD1-8u^-^[13] or its derivative strain AD∆ [14] that has been deleted of the entire *URA3* gene. Yeast strains were grown in synthetic medium (CSM pH 5.6) containing 0.077% (w/v) complete supplement mixture (Bio 101, Vista, CA), 0.67% (w/v) yeast nitrogen base without amino acids (Difco) and 2% (w/v) glucose or 2% (w/v) glycerol as carbon source or in synthetic medium buffered to pH 7.0 (CSM pH 7.0) with 10 mM 2-(*N*-morpholino)-ethanesulfonic acid (MES) and 18 mM *N*-2-hydoxyethylpiperazine-*N’*-2-ethanesulfonic acid (HEPES) or in complex YPD medium containing 1% (w/v) yeast extract, 2% (w/v) peptone, and 2% (w/v) glucose (Difco Laboratories, Detroit, MI). Yeast transformants were selected on plates containing 0.077% (w/v) complete supplement mixture without uracil (CSM–URA) (Bio 101, Vista, CA), 0.67% (w/v) yeast nitrogen base without amino acids (Difco), 2% (w/v) glucose, and 2% (w/v) agar (Difco). Plasmids were maintained in *E. coli* strain DH5α. *E. coli* cells were grown in Luria-Bertani (LB) medium, to which ampicillin was added (100 μg/ml) as required.

**Table 5:** ***Saccharomyces cerevisiae *strains used in this study**

**Strains**	**Genotype**	**Reference**
AH22	*MAT a, leu2-3, leu2-112, his 4–519, can1*	G. R. Fink, MIT, MA, USA
SY1	*MAT a, ura3-52, leu2-3, 112, his 4–619, sec6-4,* GAL	[46]
AD124567u^-^ = AD/wt-PDR5	*MAT α, PDR1-3*, *ura3*, *his1*, *∆yor1::hisG*, *∆snq2::hisG*, *∆pdr10::hisG*, *∆pdr11::hisG*, *∆ycf1::hisG*, *∆pdr3::hisG*	[13]
AD1-8u^-^ = AD	*MAT α, PDR1-3*, *ura3*, *his1*, *∆yor1::hisG*, *∆snq2::hisG*, *∆pdr10::hisG*, *∆pdr11::hisG*, *∆ycf1::hisG*, *∆pdr5::hisG*, *∆pdr15::hisG, ∆pdr3::hisG*	[13]
AD∆	AD1-8u^-^, *∆ura3*	[26]
AD/pABC3	AD1-8u^-^, *∆pdr5::*pABC3 (empty vector cassette)	[47]
AD/sec6-4	*MAT α, PDR1-3*, *ura3*, *his1*, *∆yor1::hisG*, *∆snq2::hisG*, *∆pdr10::hisG*, *∆pdr11::hisG*, *∆ycf1::hisG*, *∆pdr5::hisG*, *∆pdr15::hisG, ∆pdr3::hisG, sec6-4::200*	[26]
AD/PDR5	AD1-8u^-^, PDR5	This study
AD/SP-PDR5-URA3	AD1-8u^-^, *∆pdr5::*pABC1-SfiI-PacI-PDR5	This study
AD/S-PDR5-URA3	AD1-8u^-^, *∆pdr5::*pABC1-SfiI-PDR5	This study
AD/P-PDR5-URA3	AD1-8u^-^, *∆pdr5::*pABC3-PDR5	This study
AD/SP-PDR5	AD1-8u^-^, SfiI-PacI-PDR5	This study
AD/S-PDR5	AD1-8u^-^, SfiI(−18)-PDR5	This study
AD/P-PDR5	AD1-8u^-^, PacI-PDR5	This study
AD/sec6-4/PDR5	AD1-8u^-^, sec6-4::200, PDR5	This study
AD/sec6-4/SP-PDR5	AD1-8u^-^, sec6-4::200, SfiI-PacI-PDR5	This study
AD/sec6-4/S-PDR5	AD1-8u^-^, sec6-4::200, SfiI(−18)-PDR5	This study
AD/sec6-4/P-PDR5	AD1-8u^-^, sec6-4::200, PacI-PDR5	This study
AD/sec6-4/SP-PDR5-URA3	AD1-8u^-^, sec6-4::200, *∆pdr5::*pABC1-SfiI-PacI-PDR5	This study
AD/sec6-4/P-PDR5-URA3	AD1-8u^-^, sec6-4::200, *∆pdr5::*pABC3-PDR5	This study
AD/S-CDR2A-URA3	AD1-8u^-^, *∆pdr5::*pABC1-SfiI-CDR2A (A allele of *C. albicans* 10261)	This study
AD/P-CDR2A-URA3	AD1-8u^-^, *∆pdr5::*pABC3-CDR2A	[29]
AD/S-CDR2B-URA3	AD1-8u^-^, *∆pdr5::*pABC1-SfiI-CDR2B (B allele of *C. albicans* 10261)	This study
AD/P-CDR2B-URA3	AD1-8u^-^, *∆pdr5::*pABC3-CDR2B	[29]
AD/S-CDR1-URA3	AD1-8u^-^, *∆pdr5::*pABC1-SfiI-CaCDR1A (A allele of *C. albicans* 10261)	This study
AD∆/P-CDR1-URA3	AD∆, *∆pdr5::*pABC3-CaCDR1A	[14]
AD∆/SfiI(−4)-CDR1 = AD∆/construct1-CDR1	AD∆, *∆pdr5::*construct1	This study
AD∆/constructs(2 and 4–18)-CDR1	AD∆, *∆pdr5::*constructs(2 and 4–18)	This study

### Materials

Molecular biology reagents, restriction and modifying enzymes were from New England Biolabs (Beverly, MA) or from Roche Diagnostics N.Z. Ltd. (Auckland, New Zealand). Lyophilized desalted DNA oligonucleotides listed in Additional file 3: Table S1 were purchased from Sigma-Aldrich Pty. Ltd. (Sydney, Australia). PCR and DNA fragments were purified using kits from Qiagen Pty. Ltd. (Clifton Hill, Victoria, Australia). Genomic DNA (gDNA) was isolated from individual yeast colonies by using the Y-DER™ Yeast DNA Extraction Reagent Kit from Pierce (Rockford, IL) and downscaling the recommended protocol 50-fold. Yeast cells were transformed using the alkali-cation yeast transformation kit from Bio 101 with slight modifications for AD1-8u^-^ as described previously [14]. Plasmids and entire transformation cas-settes PCR-amplified from the gDNA of different yeast strains (Table 5) were verified by DNA sequencing using the DYEnamic ET Terminator Cycle Sequencing kit v 3.1 (Amersham Pharmacia Biotech, UK) and analyzed at the Micromon DNA Sequencing Facility (Monash University, Melbourne, Australia). For standard PCR reactions (95°C for 5 min followed by cycles of: 95°C for 20 sec; 55°C for 10 sec; and 68°C for 1 min/kb of PCR fragment) the high fidelity KOD^+^ DNA polymerase was used (Toyobo, Osaka, Japan or Novagen, San Diego, CA). For site-directed mutagenesis of plasmids the Chameleon® site-directed mutagenesis kit (Stratagene, La Jolla, CA) was employed. ExoSAP treatment was used to eliminate unwanted DNA oligomer primers from PCR reactions. In short, a 5 μl portion of the PCR reaction was incubated at 37°C with 0.2 μl ExoSAP-IT® (Affymetrix, Santa Clare, CA) for 15 min and the enzyme was heat inactivated at 80°C for 30 min. Small aliquots (0.1 – 1 μl) were then used as DNA templates for DNA sequencing or PCR.

### Compounds

Fluconazole (FLC, Diflucan; aqueous solution) was purchased from Pfizer Laboratories Ltd. (Auckland, New Zealand) and enniatin was purchased from Sigma-Aldrich New Zealand Ltd. (Auckland, New Zealand). D-octapeptide RC21v2 is a Cdr1p-specific inhibitor of FLC transport by ABC efflux pump Cdr1p [33].

### Construction of plasmids pABC1 and pABC3

Plasmid pABC1 (Figure 1A) is a pSK-PDR5-PPUS [15] derivative based on the high copy number plasmid pBluescriptIISK(+) (Stratagene). To ensure the efficient termination of highly expressed genes, the *S. cerevisiae PGK1* transcription terminator was PCR amplified as a *Hin*dIII/*Bam*HI fragment from AD1-8u^-^ gDNA and used to replace the *Hin*dIII/*Bam*HI *PDR5* terminator fragment of pSK-PDR5-PPUS immediately 3′ of the *PDR5* promoter to generate pSK-PDR5-PGK1. Further improvements (creation of a multiple cloning site with additional unique cloning sites upstream and downstream of the transformation cassette) of pSK-PDR5-PGK1 by site-directed mutagenesis led to the creation of vector pABC (precursor of pABC1).

Plasmid pABC3, the cloning vehicle that we routinely use for the overexpression of membrane proteins in yeast [14,17], was derived from plasmid pABC1 as previously described [14]. In short, we used site-directed mutagenesis to introduce a unique *Eco*RI site at the 3′ end of the *URA3* marker and replaced *Sac*I of pABC1 with *Xho*I creating plasmid pABC2. In a final step vector pABC3 was created by reverting the *Sfi*I/*Ava*I sites of pABC2 to the wildtype *PDR5* sequence to maximize translation efficiency in yeast and a second *Asc*I site was created at the 3′ end of the transformation cassette for ease of cassette excision [14].

DNA sequences of pSK-PDR5-PPUS, pSK-PDR5-PGK1, pABC, pABC1 and pABC2 were submitted to GenBank under accession numbers JN581374-78, respectively.

### Creation of *PDR5* over-expressing strains that had only their 5′ UTR modified

AD and AD/sec6-4 strains that overexpressed wt-*PDR5* or *PDR5* with their 5′ UTR modified to contain either the *Sfi*I-, the *Sfi*I-*Pac*I- or the *Pac*I-site 5′ proximal to the ATG start codon of *PDR5* were created by transforming AD and AD/sec6-4 with four different DNA fragments that contained that part of the promoter and ~1/3 (1163)bp) of the ORF of *PDR5* that was deleted in both strains [13]. To ensure proper integration of these DNA fragments via homologous recombination into the genomic *PDR5* locus of AD and AD/sec6-4 >200 bp additional *PDR5* sequence was included on either end. The DNA fragments that were used to create AD/ and AD/sec6-4/*PDR5* were PCR-amplified from gDNA of AD/wt-PDR5 using the primer pair pd5f/pd8r. The DNA fragments that were used to create AD/SP-, /S-, and /P-PDR5 and AD/sec6-4/SP-, /S-, and /P-PDR5 were obtained by digesting 2 μg of plasmids pABC1-SP-PDR5, pABC1-S-PDR5, and pABC3-PDR5, respectively, with *Asc*I and *Sal*I and gel purifying the resulting ~2.5 kb DNA fragments. Positive transformants were selected on CSM plates containing 20 μg/ml FLC, a concentration that was high enough to prevent growth of AD and AD/sec6-4 but low enough for any of the expected recombinant yeast strains to grow. Three independent transformants were verified for each individual construct for proper integration at the *PDR5* locus by PCR from purified gDNA and by DNA sequencing.

### Creation of an mRNA stem-loop library near the AUG start-codon of *CDR1*

1–10 ng of pABC3-CDR1A [14] were used as DNA template to amplify 18 pairs of PCR fragments to create 17 different yeast strains (AD∆/constructs(1,2,4-18)-CDR1; Table 4 and Additional file 2: Figure S2). Strains with weaker stem-loops (AD∆/constructs(1,2,4-8)-CDR1) were created using strategy 1, as illustrated in Additional file 2: Figure S2A. In a first step, two DNA fragments (the 3′ part of the *PDR5* promoter and the 5′ part of the *CDR1* ORF) were amplified with primers pd5f/pSfiM-(1–8)r and pSfiM-(1–8)f/Rev-3, respectively. Each pair of DNA fragments had identical stem-loop sequences near their 3′ (*PDR5* fragment) and 5′ (5′-*CDR1* fragment) ends, respectively (highlighted light blue in Additional file 1: Figure S2 and underlined sequences in Additional file 3: Table S1). Portions of PCR amplicons were treated with ExoSAP-IT® to eliminate DNA oligomer primers before mixing 1 μl (~40 ng) of each pair of DNA fragments (construct pairs 1–8) and amplifying the fused PCR products with the outside primer pair pd5f/Rev-3 in a second PCR step. The eight fused PCR products were column purified and used to transform AD∆ as described below. Using this approach it was impossible to amplify the fused PCR product for AD∆/construct3-CDR1 (core *Sfi*I stem-loop extended with three extra GC-pairs; see primers pSfiM-3f/r in Additional file 3: Table S1). This strong stem-loop of seven GC-pairs inhibited the fusion of the two overlapping PCR fragments as illustrated in Additional file 2: Figure S2B (top left).

In order to create larger stem-loops with additional AU- and GC-pairs (including AD∆/construct15-CDR1 to replace the planned but not obtainable AD∆/construct3-CDR1) we developed an alternative cloning strategy (Additional file 2: Figure S2B). Primer pairs pSfiM-(9–18)(f/r) were designed so that their 5′ ends contained a core *Sfi*I site (bold type face; Additional file 3: Table S1) that was extended on either side with one, two, or three extra nucleotides (underlined in Additional file 3: Table S1). The sequences for primers pSfiM-(9–18)f were extended with three additional As followed by ~20 bp of the *CDR1A* ORF. This design ensured that the positions of the stem-loops of constructs 9–18 were always at −4 relative to the AUG start-codon. The sequences of primers pSfiM-(9–18)r were each extended with an additional three Ts so that each sequence of the *Sfi*I constructs(9–18) was flanked by three A nucleotides (Additional file 3: Table S1) to minimize secondary structure around the predicted stem-loops. An additional ~25 bp of the wild-type *PDR5* promoter sequence was added to the reverse primers to ensure the amplification of PCR products. The *PDR5* promoter and the 5′-*CDR1* fragments were then amplified by PCR from pABC3-CDR1A with pd5f/pSfiM-(9–18)r and pSfiM-(9–18)f/Rev-3 primer pairs, respectively. A portion (~200 ng) of each PCR fragment was digested with *Sfi*I, the digested fragments were then gel purified and dissolved in 50 μl water. Corresponding pairs of *Sfi*I-digested PCR fragments (5 μl; ~20 ng) were mixed, ligated, and ~2 ng of each ligation mix (mixes 9–18) used as a DNA template for PCR amplification of ligated products using primers pd5f/Rev-3 (Additional file 2: Figure S2B). A single PCR fragment was obtained in all cases. This was possible for two reasons: i) the two DNA fragments of each pair of PCR fragments could only ligate at their *Sfi*I digested ends (grey) leading to only three possible ligation products (Additional file 2: Figure S2B) as their blunt ends were not 5′-phosphorylated (non-phosphorylated primers were used to amplify the fragments); and ii) only fragment three of the ligation mix (*PDR5*-*Sfi*I-5′-*CDR1*; Additional file 1: Figure S2B) could be amplified because the other two ligation products were inverted repeats that spontaneously form an intramolecular double strand after strand separation at 94°C.

Finally, the fused and PCR amplified DNA products obtained by either of these two strategies (Additional file 2: Figure S2A and B) were gel purified, and ~40 ng of each were mixed with 200 ng 3′-*CDR1*-*URA3*-*PDR5* (PCR amplified from pABC3-CDR1A with primers CaCDR1-3/pAscI-2 and column purified; the remaining 3′ part of the entire *CDR1*-transformation cassette was identical for all constructs) and used to transform AD∆ (Additional file 2: Figure S2C). The entire *CDR1*-transformation cassette (~7.5 kb) was divided into two parts (5′ *CDR1* and 3′ *CDR1*) because the smaller (~2.5 kb) 5′ *CDR1* constructs required fewer cycles (a combined total of 40–45 cycles for two separate steps of PCR amplification required for either strategy; Additional file 2: Figure S2A and B) of PCR to efficiently amplify, which significantly reduced the rate of amplification errors within positive transformants. Uracil prototroph transformants were selected on CSM-URA plates after incubation at 30°C for 2–3 d. Three independent transformants were verified for each individual construct for proper integration at the *PDR5* locus by PCR from purified gDNA and by DNA sequencing.

### Northern blot analysis

Total RNA was isolated from *S. cerevisiae* cells using the hot-phenol extraction method. Usually about 100 ODU (optical density units; defined as the amount of cells corresponding to 1 ml of cells of an OD_600_ of 1) of cells were harvested by centrifugation for 1 min at 3000 g, the cells were washed once in ice-cold water, and snap frozen in liquid nitrogen and stored at −80°C. Samples (10 μg) of total RNA were separated on 1.2% denaturing agarose gels and stained with ethidium bromide (EtBr). The separated total RNA was photographed, immediately Northern blotted onto nylon^+^ membranes and further processed according to standard protocols [48]. ^32^P-radioactively labeled probes for *ACT1* and *PDR5* were obtained with a random priming kit using PCR-amplified DNA fragments of *ACT1* (~800 bp; amplified with pACT1for/pACT1rev) and *PDR5* (~1200 bp; amplified with pd7f/pd23r) as DNA templates. Both PCR fragments were amplified from gDNA extracted from AD/wt-PDR5.

### Analysis and purification of plasma membrane (PM) proteins

PM fractions of *S. cerevisiae* cells were prepared as described previously [49] and protein samples (30 μg) were separated by SDS-PAGE with 8% polyacrylamide gels and stained with Coomassie Blue R250.

### Functional analysis of multidrug efflux pump over-expressing yeast strains

The susceptibilities of three independent transformants for each individual construct to the antifungal FLC were measured as described previously [49].

### Screening for inhibitors of *C. albicans* multidrug efflux pump Cdr1p (chemosensitization assay)

The chemosensitization of yeast strains over-expressing the *C. albicans* multidrug efflux pump Cdr1p to FLC was carried out as described previously [49]. In brief, a 10 ml YPD overnight culture of cells was diluted 1:20 into CSM medium and incubated at 30°C for a further four hours. Each test strain (OD_600nm_ ~1) was diluted to OD_600nm_ = 0.008 in 5 ml of melted CSM containing 0.6% agarose (50°C) and FLC at 0.25 x the minimum growth inhibitory concentration (MIC_FLC_) of each strain. The cell suspension was poured into a rectangular Omnitray plate (126 by 86 by 19 mm; Nunc, Roskilde, Denmark) that contained 20 ml of CSM solidified with 0.6% agarose and FLC at a concentration of 0.25 x MIC_FLC_ of the respective test strain. Whatman 3MM paper disks containing different amounts of the Cdr1p drug pump inhibitor enniatin or RC21v2 were placed on the solidified top agarose and the plates were incubated at 30°C for 48 h.

## Competing interests

The authors declare that they have no competing interests.

## Authors’ contributions

EL, MN, and RDC contributed to the design of the study and EL performed the majority of the experiments. All authors participated in writing and critical review of the manuscript. All authors have read and approved the manuscript.

## Supplementary Material

Additional file 1: Figure S1 Northern blot analysis (Additional file 1: Figure S1) was performed on cells harvested at early log (OD_600_ = 0.5), late log (OD_600_ = 5) or stationary phase (OD_600_ = 10–12; these cells were grown in CSM (pH 5.6) for an additional 16 h compared to late-log phase cells) as described in the Materials and methods section. The experiment was performed on AD/sec6-4/PDR5, AD/sec6-4/S- and /P-PDR5 and two wild-type *PDR5* expressing strains, AH22 and SY1. Comparing the measured intensities between the short- and long-exposed bands for *PDR5* (Additional file 1: Figure S1A) that were within the linear range in both autoradigraphs helped determine the factor (18.3) by which the bottom autoradiograph was overexposed. This factor was used to analyze the Northern blot results (Additional file 1: Figure S1A) as presented in Additional file 1: Figure S1B-D. The *Sfi*I mRNA stem-loop near the AUG start codon causes ∼3-fold increased mRNA levels of *PDR5* that is independent of the growth phase. **A** shows the two large ribosomal bands of total RNA extracts after they had been separated with a 1.2% denaturing agarose gel and stained with EtBr (top). Total RNA was extracted from the indicated strains harvested either at early log-, late log-, or stationary phase (from left to right), respectively. As in Figure 2A, the autoradiograph obtained for *PDR5* and *ACT1* is shown underneath, and an overexposed autoradiograph for *PDR5* is shown at the very bottom. **B** shows the growth-phase dependent change in *ACT1* mRNA levels relative to early log phase cells for the five test strains (*ACT1* mRNA levels of early log phase cells are shown as black bars, late log phase cells as dark grey bars and stationary phase cells as light grey bars). **C** shows the growth-phase dependent change in *PDR5* mRNA levels relative to early log phase cells for the same strains and using the same assignment of bars as in **B** for early log, late log, and stationary phase cells. **D** shows the change in normalized (i.e. relative to *ACT1*) *PDR5* mRNA levels for all five test strains harvested at early log (black bars), late log (dark grey bars) or stationary growth phase (light grey bars), and **E** shows the -fold differences of the normalized *PDR5* mRNA levels relative to AD/sec6-4/PDR5 harvested for early log (0.5), late log (5) or stationary (10) phase cells, respectively (dark grey bars = wt-PDR5; black bars = S-PDR5 and light grey bars = P-PDR5, respectively). The numbers above individual bars in **B, C, D,** and **E** give the actual values represented by the bars. Click here for file

Additional file 2: Figure S2 Two simple PCR-based cloning strategies to create AD∆ strains over-expressing *CDR1* with either weak or strong GC-rich mRNA stem-loops at position −4. **A.** Strategy 1: The seven *PDR5* promoter fragments (PDR5p; green) including the *Sfi*I stem-loop structure at its 3′ end (light blue); and seven fragments comprising ~1/3 of the *CDR1* ORF (*5′ CDR1*; orange) including 25 bp at its 5′ end that overlap with their respective *PDR5*-fragments (light blue) were amplified by PCR. All 14 fragments were treated with ExoSAP-IT® to eliminate excess primers and equimolar amounts of these 7 PDR5p/5′ *CDR1* fragment pairs were mixed and amplified by fusion PCR with primers pd5f/Rev-3. The fused PCR fragments *5′ CDR1(1,2,4-8)* were column purified and used to transform AD∆ as shown in **C. B.** The stronger stem-loop-constructs *5′ CDR1(9–18)* were created with strategy 2 because these strong stem-loops prevented the fusion of respective PCR fragment pairs (top left). Each pair of these strong stem-loop-containing PCR fragments was amplified with the indicated primers (top). Aliquots of each PCR were digested with *Sfi*I and gel purified. Equimolar amounts of each pair of *Sfi*I-digested PCR fragments were ligated and aliquots (grey box in the middle) were then used to PCR amplify the fused fragments with primers pd5f/Rev-3 (*Sfi*I stem-loop sequences are highlighted in light blue). **C.** The 17 different 5′-*CDR1(1,2,4-18)* fragments obtained in **A** and **B** were mixed in equimolar amounts with the PCR amplified (from pABC3-CDR1), and column purified, *3′ CDR1* transformation cassette (~2/3 of *3′ CDR1* (orange) - *PGK1* terminator (blue) - *URA3* selection marker (purple) - and *PDR5*downstream (green)) and used to transform AD∆ to Ura^+^, creating strains expressing 17 different *CDR1* mRNA stem-loop constructs. Correct integration of the transformation cassettes at the *PDR5* locus required a triple homologous cross-over event (indicated by crosses) that was confirmed by PCR and DNA sequencing. Click here for file

Additional file 3: Table S1 DNA oligomer primers used in this study. Click here for file
