# Supplementary material for: Small, synthetic, GC-rich mRNA stem-loop modules 5′ proximal to the AUG start-codon predictably tune gene expression in yeast
Source: Microb Cell Fact. 2013 Jul 29;12:74. doi: 10.1186/1475-2859-12-74 (PMC3765126; doi:10.1186/1475-2859-12-74)
Supplement: Additional file 1: Figure S1 — Northern blot analysis (Additional file 1: Figure S1) was performed on cells harvested at early log (OD600 = 0.5), late log (OD600 = 5) or stationary phase (OD600 = 10–12; these cells were grown in CSM (pH 5.6) for an additional 16 h compared to late-log phase cells) as described in the Materials and methods section. The experiment was performed on AD/sec6-4/PDR5, AD/sec6-4/S- and /P-PDR5 and two wild-type PDR5 expressing strains, AH22 and SY1. Comparing the measured intensities between the short- and long-exposed bands for PDR5 (Additional file 1: Figure S1A) that were within the linear range in both autoradigraphs helped determine the factor (18.3) by which the bottom autoradiograph was overexposed. This factor was used to analyze the Northern blot results (Additional file 1: Figure S1A) as presented in Additional file 1: Figure S1B-D. The SfiI mRNA stem-loop near the AUG start codon causes ∼3-fold increased mRNA levels of PDR5 that is independent of the growth phase. A shows the two large ribosomal bands of total RNA extracts after they had been separated with a 1.2% denaturing agarose gel and stained with EtBr (top). Total RNA was extracted from the indicated strains harvested either at early log-, late log-, or stationary phase (from left to right), respectively. As in Figure 2A, the autoradiograph obtained for PDR5 and ACT1 is shown underneath, and an overexposed autoradiograph for PDR5 is shown at the very bottom. B shows the growth-phase dependent change in ACT1 mRNA levels relative to early log phase cells for the five test strains (ACT1 mRNA levels of early log phase cells are shown as black bars, late log phase cells as dark grey bars and stationary phase cells as light grey bars). C shows the growth-phase dependent change in PDR5 mRNA levels relative to early log phase cells for the same strains and using the same assignment of bars as in B for early log, late log, and stationary phase cells. D shows the change in normalized (i.e. relative to ACT1) PDR5 [file 1475-2859-12-74-S1.pptx]

## Slide 1
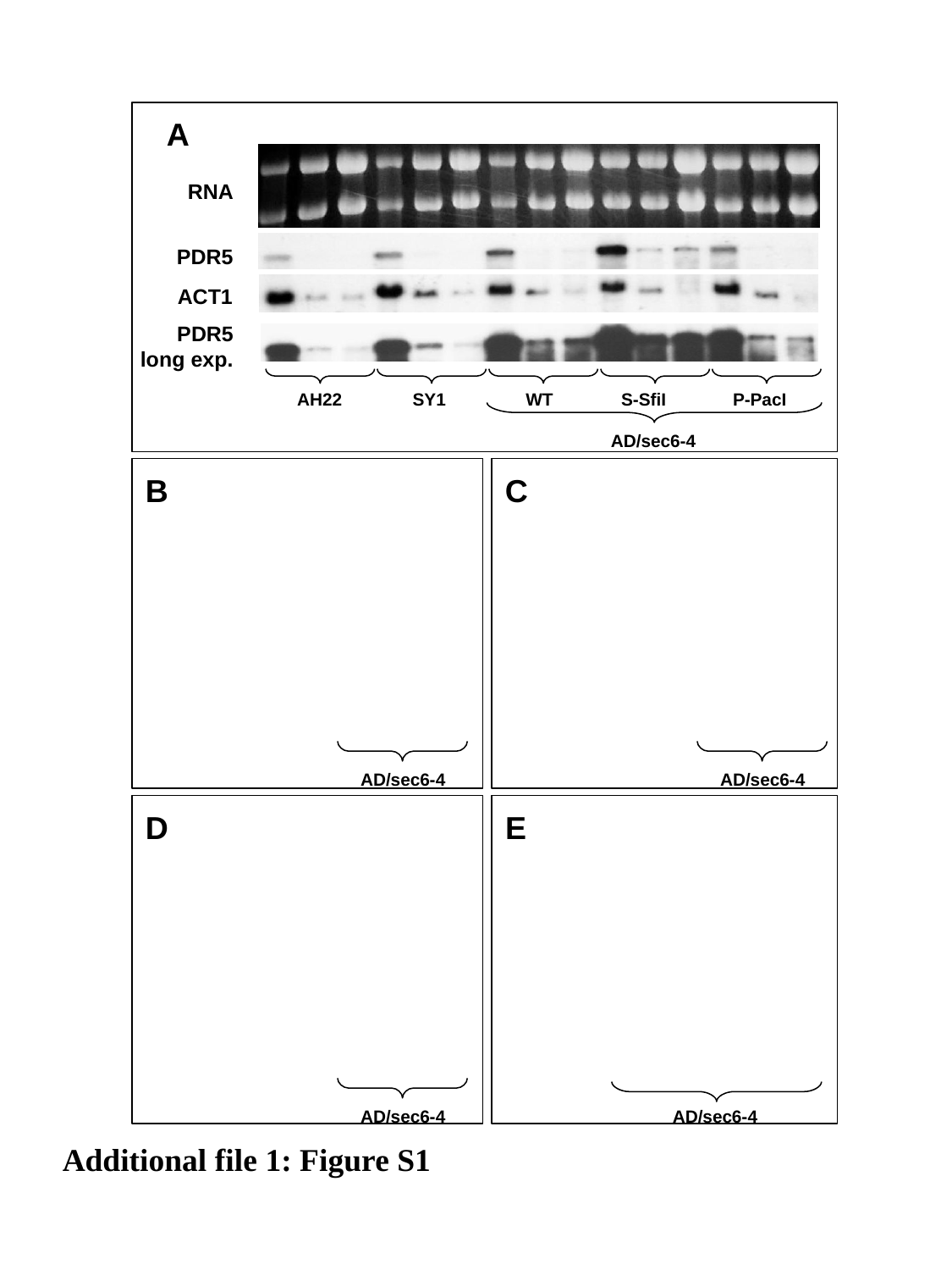

A
RNA
PDR5
ACT1
PDR5
long exp.
AH22
SY1
WT
S-SfiI
P-PacI
AD/sec6-4
B
C
AD/sec6-4
AD/sec6-4
D
E
AD/sec6-4
AD/sec6-4
Additional file 1: Figure S1
