# Supplementary material for: Small, synthetic, GC-rich mRNA stem-loop modules 5′ proximal to the AUG start-codon predictably tune gene expression in yeast
Source: Microb Cell Fact. 2013 Jul 29;12:74. doi: 10.1186/1475-2859-12-74 (PMC3765126; doi:10.1186/1475-2859-12-74)
Supplement: Additional file 2: Figure S2 — Two simple PCR-based cloning strategies to create AD∆ strains over-expressing CDR1 with either weak or strong GC-rich mRNA stem-loops at position −4. A. Strategy 1: The seven PDR5 promoter fragments (PDR5p; green) including the SfiI stem-loop structure at its 3′ end (light blue); and seven fragments comprising ~1/3 of the CDR1 ORF (5′ CDR1; orange) including 25 bp at its 5′ end that overlap with their respective PDR5-fragments (light blue) were amplified by PCR. All 14 fragments were treated with ExoSAP-IT® to eliminate excess primers and equimolar amounts of these 7 PDR5p/5′ CDR1 fragment pairs were mixed and amplified by fusion PCR with primers pd5f/Rev-3. The fused PCR fragments 5′ CDR1(1,2,4-8) were column purified and used to transform AD∆ as shown in C. B. The stronger stem-loop-constructs 5′ CDR1(9–18) were created with strategy 2 because these strong stem-loops prevented the fusion of respective PCR fragment pairs (top left). Each pair of these strong stem-loop-containing PCR fragments was amplified with the indicated primers (top). Aliquots of each PCR were digested with SfiI and gel purified. Equimolar amounts of each pair of SfiI-digested PCR fragments were ligated and aliquots (grey box in the middle) were then used to PCR amplify the fused fragments with primers pd5f/Rev-3 (SfiI stem-loop sequences are highlighted in light blue). C. The 17 different 5′-CDR1(1,2,4-18) fragments obtained in A and B were mixed in equimolar amounts with the PCR amplified (from pABC3-CDR1), and column purified, 3′ CDR1 transformation cassette (~2/3 of 3′ CDR1 (orange) - PGK1 terminator (blue) - URA3 selection marker (purple) - and PDR5downstream (green)) and used to transform AD∆ to Ura+, creating strains expressing 17 different CDR1 mRNA stem-loop constructs. Correct integration of the transformation cassettes at the PDR5 locus required a triple homologous cross-over event (indicated by crosses) that was confirmed by PCR and DNA sequencing. [file 1475-2859-12-74-S2.pptx]

## Slide 1
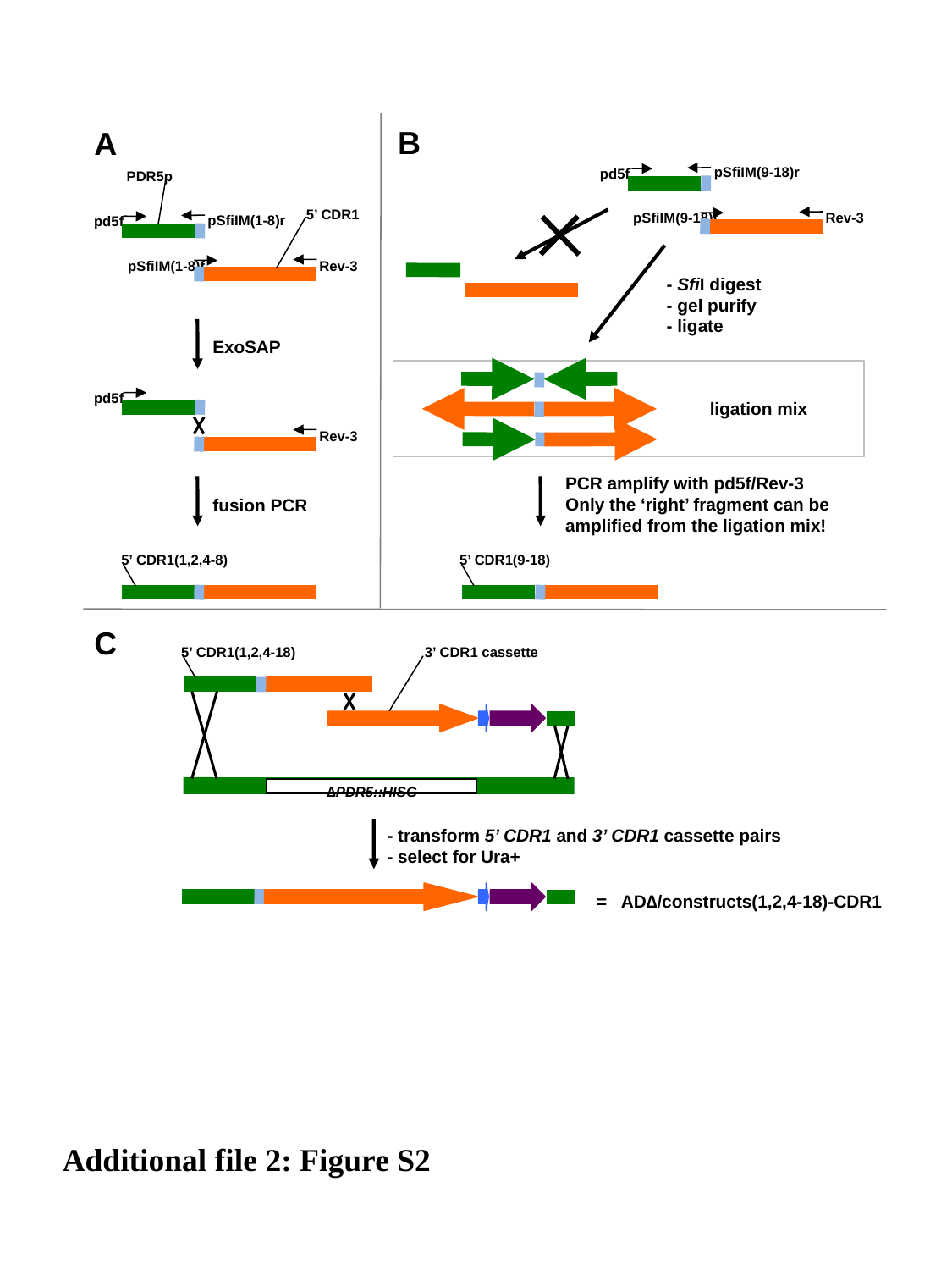

B
A
pSfiIM(9-18)r
pd5f
PDR5p
5’ CDR1
pSfiIM(9-18)f
Rev-3
pSfiIM(1-8)r
pd5f
pSfiIM(1-8)f
Rev-3
- SfiI digest
- gel purify
- ligate
ExoSAP
pd5f
ligation mix
Rev-3
PCR amplify with pd5f/Rev-3
Only the ‘right’ fragment can be amplified from the ligation mix!
fusion PCR
5’ CDR1(1,2,4-8)
5’ CDR1(9-18)
C
5’ CDR1(1,2,4-18)
3’ CDR1 cassette
∆PDR5::HISG
- transform 5’ CDR1 and 3’ CDR1 cassette pairs
- select for Ura+
= AD∆/constructs(1,2,4-18)-CDR1
Additional file 2: Figure S2
