# Supplementary material for: Small, synthetic, GC-rich mRNA stem-loop modules 5′ proximal to the AUG start-codon predictably tune gene expression in yeast
Source: Microb Cell Fact. 2013 Jul 29;12:74. doi: 10.1186/1475-2859-12-74 (PMC3765126; doi:10.1186/1475-2859-12-74)
Supplement: Additional file 3: Table S1 — DNA oligomer primers used in this study. [file 1475-2859-12-74-S3.doc]

**Additional file 3: Table S1: DNA oligomer primers used in this study.**

| Oligonucleotides | Sequences, 5’ – 3’ |
| --- | --- |
| SfiIPacI-PDR5 | GTTTTCGTGGCCGCTCGGGCCAAAGACTTAATTAAAAA*ATG*CCCGAGGC |
| SfiI-PDR5 | GTTTTCGTGGCCGCTCGGGCCAAAGACTTTAG |
| NotI-PDR5 | AGTGCGGCCGCAT*TTA*TTTCTTGGAGAGTTTACCGTTCTTT |
| PacI-CDR2 | GACTTATTAAAAA*ATG*AGTACTGCAAACACGTCTTTG |
| SfiI-CDR2 | TTTCGTGGCCGCTCGGGCCAAAGACTTTAGACAAAA*ATG*AGTACTGCAAACACGTCTTTG |
| NotI-CDR2 | GAAGCGCCGCCC*TTA*TTTTTTCATCTTCTTTTCTCTATTACC |
| PacI-CDR1 | AAATTAATTAAAAA*ATG*TCAGATTCTAAGATGTCG |
| SfiI-CDR1 | CGTGGCCGCTCGGGCCAAAGACTTTAGACAAAA*ATG*TCAGATTCTAAGATGTC |
| NotI-CDR1 | AAAGCGGCCGCAT*TTA*TTTCTTATTTTTTTTCTCTCTG |
| pAscI-2 | GCCGGCCGCACTAGACTTGGCGCGCCTACCGTTCTTTTTAGGC |
| pd5f | gaacatgaacgttcctcagcgcg |
| pd7f | GTTGTTTACAACGCAGAAGCTG |
| pd8r | CATCGTCATTCAATAATCGTTGG |
| pd23r | GTAGAAAAAGAATACACCAC |
| pACT1for | TGGCATCATACCTTCTACAACG |
| pACT1rev | AAGAAGCCAAGATAGAACCACC |
| Rev3 | CGAAGGAAATTTCTCGCAAC |
| CaCDR1-3 | GGGTTAGATTCTGCTACTGC |
| psfiM-1f | **CCGCTCGTTCAGGCCGCTCGGGCCT**AAAATGTCAGATTCTAAGATGTCGT |
| psfiM-1r | **AGGCCCGAGCGGCCTGAACGAGCGG**ATACGAAAACTT |
| psfiM-2f | **CCGCTCGTTCCGGCCGCTCGGGCCG**AAAATGTCAGATTCTAAGATGTCGT |
| psfiM-2r | **CGGCCCGAGCGGCCGGAACGAGCGG**ATACGAAAACTT |
| psfiM-3f | **CCGCTCCGCGGCCGCTCGGGCCGCG**AAAATGTCAGATTCTAAGATGTCGT |
| psfiM-3r | **CGCGGCCCGAGCGGCCGCGGAGCGG**ATACGAAAACTTAAAAG |
| psfiM-4f | **CCGCTCGTTCGAGGCCGCTCGGGCG**AAAATGTCAGATTCTAAGATGTCGT |
| psfiM-4r | **CGCCCGAGCGGCCTCGAACGAGCGG**ATACGAAAAC |
| psfiM-5f | **CCGCTCGTTCGATTCCGCTCGGGCC**AAAATGTCAGATTCTAAGATGTCGT |
| psfiM-5r | **GGCCCGAGCGGAATCGAACGAGCGG**ATACGAAAAC |
| psfiM-6f | **CCGCTCGAGGCCAAGCTCGAGGCCT**AAAATGTCAGATTCTAAGATGTCGT |
| psfiM-6r | **AGGCCTCGAGCTTGGCCTCGAGCGG**ATACGAAAACTTAAAAG |
| psfiM-7f | **CCGCTCGTTCGAAAGGCCAAGGCCT**AAAATGTCAGATTCTAAGATGTCGT |
| psfiM-7r | **AGGCCTTGGCCTTTCGAACGAGCGG**ATACGAAAAC |
| psfiM-8f | **CCGCTCGTTCGAAAGAGGCCGGCCT**AAAATGTCAGATTCTAAGATGTCGT |
| psfiM-8r | **AGGCCGGCCTCTTTCGAACGAGCGG**ATACGAA |
| psfiM-9f | AAA**GGCCGCTCGGGCC**AAAAATGTCAGATTCTAAGATGTCGT |
| psfiM-9r | TTT**GGCCCGAGCGGCC**TTTTCGAGCGGATACGAAAACTTAAAAG |
| psfiM-10f | AAT**GGCCGCTCGGGCC**AAAAATGTCAGATTCTAAGATGTCGT |
| psfiM-10r | TTT**GGCCCGAGCGGCC**ATTTCGAGCGGATACGAAAACTTAAAAG |
| psfiM-11f | AAC**GGCCGCTCGGGCC**GAAAATGTCAGATTCTAAGATGTCGT |
| psfiM-11r | TTC**GGCCCGAGCGGCC**GTTTCGAGCGGATACGAAAACTTAAAAG |
| psfiM-12f | AAGA**GGCCGCTCGGGCC**TCAAAATGTCAGATTCTAAGATGTCGT |
| psfiM-12r | TTGA**GGCCCGAGCGGCC**TCTTTAGCGGATACGAAAACTTAAAAGGG |
| psfiM-13f | AATA**GGCCGCTCGGGCC**TAAAAATGTCAGATTCTAAGATGTCGT |
| psfiM-13r | TTTA**GGCCCGAGCGGCC**TATTTAGCGGATACGAAAACTTAAAAGGG |
| psfiM-14f | AAGC**GGCCGCTCGGGCC**GCAAAATGTCAGATTCTAAGATGTCGT |
| psfiM-14r | TTGC**GGCCCGAGCGGCC**GCTTTAGCGGATACGAAAACTTAAAAGGG |
| psfiM-15f | AACGC**GGCCGCTCGGGCC**GCGAAAATGTCAGATTCTAAGATGTCGT |
| psfiM-15r | TTCGC**GGCCCGAGCGGCC**GCGTTTCGGATACGAAAACTTAAAAGGGTC |
| psfiM-16f | AACGA**GGCCGCTCGGGCC**TCGAAAATGTCAGATTCTAAGATGTCGT |
| psfiM-16r | TTCGA**GGCCCGAGCGGCC**TCGTTTCGGATACGAAAACTTAAAAGGGTC |
| psfiM-17f | AATGA**GGCCGCTCGGGCC**TCAAAAATGTCAGATTCTAAGATGTCGT |
| psfiM-17r | TTTGA**GGCCCGAGCGGCC**TCATTTCGGATACGAAAACTTAAAAGGGTC |
| psfiM-18f | AATTA**GGCCGCTCGGGCC**TAAAAAATGTCAGATTCTAAGATGTCGT |
| psfiM-18r | TTTTA**GGCCCGAGCGGCC**TAATTTCGGATACGAAAACTTAAAAGGGTC |

*Sfi*I, *Pac*I and *Not*I sites are underlined and the start and stop codons of primers used to amplify heterologous ORFs are highlighted in italics. psfiM-1f to psfiM-18r are 18 primer pairs (f = forward; r = reverse) that were used to create 18 modified *Sfi*I stem-loop constructs. Underlined sequences of the top ten primers indicate the recognition sites for the indicated restriction enzymes. Bold type letters for the psfiM-1-9f/r primers pairs indicate the 25 complementary nucleotides that created 25 bp overlaps (blue in Supplementary Fig. 1 A) between individual primer pairs, and the underlined sequences for the psfiM-1-9f/r primer pairs indicate the modified *Sfi*I stem-loop sequences. Bold type letters for the psfiM-10-19f/r primer pairs indicate the core *Sfi*I stem-loop sequences that were extended on either end (underlined sequences) with complementary sequences to create the larger GC-pair stem-loop constructs 10-18 (Supplementary Fig. 1 B).
